# A Ligand-Free Approach towards Coumarin Analogs via Natural Deep Eutectic Solvent-Mediated Suzuki–Miyaura Coupling

**DOI:** 10.3390/molecules29184398

**Published:** 2024-09-16

**Authors:** Annita Katopodi, Nikolaos Nikolaou, Vasiliki Kakokefalou, Eleni Alexandratou, Manolis Matzapetakis, Maria Zervou, Anastasia Detsi

**Affiliations:** 1 Laboratory of Organic Chemistry, School of Chemical Engineering, National Technical University of Athens, Zografou Campus, 15780 Athens, Greece; annitakatopodi@mail.ntua.gr (A.K.); nickosnikolaou@yahoo.gr (N.N.); vasilikikakokefalou@mail.ntua.gr (V.K.); 2 Laboratory of Biomedical Optics and Applied Biophysics, School of Electrical and Computer Engineering, National Technical University of Athens, Zografou Campus, 15780 Athens, Greece; ealexan@central.ntua.gr; 3 Institute of Chemical Biology, National Hellenic Research Foundation, 48 Vas. Constantinou Ave., 11635 Athens, Greece; matzman@eie.gr (M.M.); mzervou@eie.gr (M.Z.)

**Keywords:** Suzuki–Miyaura reaction, Deep Eutectic Solvents, coumarins, palladium nanoparticles

## Abstract

A ligand-free approach for the Suzuki-Miyaura cross coupling reaction using Natural Deep Eutectic Solvents (NaDES) towards coumarin analogs is described. A model reaction between the synthetically prepared 3-(4-acetyloxy-phenyl)-6-bromo-4-methyl-coumarin (**3b**) and phenylboronic acid was performed in five different NaDES as well as in pure glycerol, using two inorganic bases and palladium catalysts. The reaction proceeded smoothly in Choline Chloride/Glycerol (ChCl/Gly) and Betaine/Glycerol (Bet/Gly) NaDES at 90 °C in 24 h, affording the desired product in high yields up to 95%. The combination of K_2_CO_3_, Pd(OAc)_2_ and ChCl/Gly NaDES provided optimum yields and high purity of the desired compounds, while the solvent was successfully recycled and reused up to two times. The developed methodology is applicable to boronic acids bearing various substituents. The formation of palladium nanoparticles in the reaction mixture was observed, and the size of the nanoparticles was associated with the reaction yield. In addition, in all the glycerol-based NaDES, an effective removal of the acetyl group of the acetyloxy–coumarin analogs was observed; thus, it is noteworthy that the Suzuki–Miyaura coupling and the deacetylation reaction were achieved in one pot. The ten novel coumarin derivatives synthesized were structurally characterized using 1D and 2D NMR spectroscopy and were tested for their cytotoxicity against the A431 squamous cancer cell line, presenting significant activity.

## 1. Introduction

Deep eutectic solvents (DES) are liquid eutectic mixtures of at least two components, a hydrogen bond donor and a hydrogen bond acceptor, in appropriate molar ratios, which are capable of forming intermolecular interactions. Natural deep eutectic solvents (NaDES) are composed of naturally occurring metabolites; they are biodegradable and present chemical and thermal stability [1,2]. NaDES have emerged as a promising alternative to conventional organic solvents, finding applications in various scientific fields such as extraction and catalysis [3]. NaDES have also been recently used as reducing and stabilizing agents for the synthesis of metallic nanoparticles (NPs) [4].

Suzuki–Miyaura coupling is one of the most commonly used carbon–carbon bond-forming reactions in the pharmaceutical industry. Conventionally, the reaction occurs between aryl boronic acid and an aryl halide in the presence of a palladium catalyst and an inorganic base in a suitable solvent, such as toluene and dioxane [5]. The palladium catalyst is usually partnered with a ligand that stabilizes the metallic species and facilitates the reaction, such as phopshines. However, phosphine-free or ligand-free catalytic systems are more cost-effective and environmentally friendly and thus have received increasing interest [6,7,8]. Moreover, the use of common organic solvents has to be diminished in order to implement more sustainable synthetic procedures. In this context, glycerol has been explored by Wolfson and Dlugy in 2006 [9] as an alternative, biodegradable, and greener solvent for Suzuki–Miyaura coupling using the reaction between aryl halides and phenyl boronic acid as the model reaction. The group of Cravotto et al. [10] have shown the utility of glycerol in the same reaction, using ultrasound or microwave heating, whereas very recently, Monteiro et al. [11] used glycerol and a glycerol–triethanolamine deep eutectic solvent to perform the Pd-catalyzed Suzuki–Miyaura cross-coupling of aryl halides with arylboronic acids.

Imperato et al., in 2006 [12], studied the Suzuki–Miyaura coupling reaction between phenyl boronic acid and three different aryl bromides in low melting mixtures consisting of sugars, urea and inorganic salts as a solvent, Pd(OAc)_2_ as a catalyst and Na_2_CO_3_ as the base. This is considered to be the first report on Suzuki–Miyaura coupling in NaDES, which was followed by several other interesting research works on the NaDES as solvents for this reaction [13,14,15,16]. Since then, several glycerol-containing NaDES have been applied as solvents for Suzuki–Miyaura cross coupling [17,18]. In the majority of cases, the choline chloride/glycerol 1:2 (molar ratio) NaDES seems to work better in the model reaction between aryl halides and phenyl boronic acid or potassium aryltrifluoroborates [19,20,21]. Moreover, in 2023, Thiery et al. [22] presented a mechanochemical approach using choline chloride/glycerol 1:2 (molar ratio) NaDES as a grinding additive for the effective synthesis of biaryls via the Suzuki–Miyaura cross-coupling reaction, with good yields, short reaction times and no additional heating or ligand.

Coumarins constitute a large class of naturally or synthetically occurring heterocyclic compounds, which belong to the benzopyrone family and possess a wide array of biological activities including antioxidant, anti-inflammatory, neuroprotective and anticancer activities [23]. Based on our previous research as well as recent literature, aryl coumarins present significant bioactivities, and thus, several methodologies have been developed regarding their synthesis [24,25]. The insertion of an aryl or a heteroaryl moiety on the coumarin scaffold can also be achieved using Suzuki–Miyaura coupling. For instance, in the work of Roussaki et al. [26], the insertion of an aryl moiety at position 6 of the coumarin scaffold was achieved using Cs_2_CO_3_ and Pd(PPh_3_)_4_ in dimethylformamide under reflux. In addition, in the work of Hamdy et al. [27], the arylation of the coumarin derivatives was achieved via Suzuki–Miyaura coupling using K_3_PO_4_ and Pd(PPh_3_)_4_ in 1,4-dioxane at 120 °C for 6 h. However, to the best of our knowledge, no approaches for Suzuki–Miyaura coupling based on the coumarin scaffold using ligand-free and/or green solvents have been reported.

Therefore, in continuation of our previous studies on the use of NaDES as solvents and catalysts for organic reactions, we set out to synthesize a series of multi-substituted coumarins via the Suzuki–Miyaura coupling reaction. In an effort to expand our “chemical library” of coumarin derivatives bearing various substituents on the benzopyrone moiety, the 6-bromo-coumarin scaffold was selected as a substrate, along with different aryl and heteroaryl boronic acids, palladium catalysts and inorganic bases and NaDES as solvents. A model reaction between 3-(4-acetyloxy-phenyl)-6-bromo-4-methyl-coumarin (**3b**) and phenyl boronic acid was selected to perform the optimization of the synthetic process, while the recyclability and reusability of the NaDES was also assessed. The interesting observation concerning the formation of palladium nanoparticles during the reaction was also further investigated. The novel coumarin analogs that were synthesized were examined for their cytotoxicity against the A431 squamous carcinoma cell line.

## 2. Results and Discussion

### 2.1. Optimization of the Reaction Protocol

In an effort to develop a ligand-free and organic solvent-free approach for the synthesis of multi-substituted coumarins via Suzuki–Miyaura coupling, different NaDES as well as pure glycerol were used as solvents, along with sodium carbonate (Na_2_CO_3_) and potassium carbonate (K_2_CO_3_) as bases and palladium chloride (PdCl_2_) and palladium acetate (Pd(OAc)_2_) as catalysts (Table 1).

**Scheme 1:**
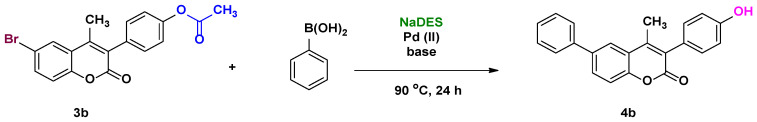
Model reaction: The reaction was performed by adding **3b**, phenylboronic acid and the appropriate NaDES, base and Pd(II) catalyst in a round bottomed flask and heating at 90 °C for 24 h.

The optimization of the methodology for the synthesis of the aryl-coumarins was performed using a model reaction between the synthetically prepared 3-(4-acetyloxy-phenyl)-6-bromo-4-methyl-coumarin **3b** [24] and phenyl boronic acid (Scheme 1).

It can be observed that the reaction proceeded smoothly after 24 h in both Choline chloride/Glycerol (ChCl/Gly) = 1:2 and Betaine/Glycerol (Bet/Gly) = 1:2 NaDES, and the product was obtained in high yields (70–95%). In the case of L-proline/Glycerol (Pro/Gly) = 1:2 NaDES as well as in pure glycerol, only traces of the product were obtained. It is noteworthy that, when the non-glycerol-containing NaDES Fructose/Urea/H_2_O = 1:1.5:1 and Glucose/Urea/H_2_O = 1:1.5:1 were used, the reaction did not proceed at all. A plausible explanation is that the monosaccharide-containing NaDES were very viscous and did not allow for the adequate mixing and solubilization of the reactants. The highest yield was obtained by using Choline chloride/Glycerol = 1:2 NaDES as a solvent along with K_2_CO_3_ as a base and Pd(OAc)_2_ (0.025 mol/mol Pd(II)/**3b**) as a catalyst, affording the product in a 95% yield.

Apart from the successful C-C formation reaction in the developed conditions, the concomitant removal of the acetyl group of compound **3b** was observed in all the glycerol-based NaDES, leading to the deacetylated final product (**4b**).

**Figure 1:**
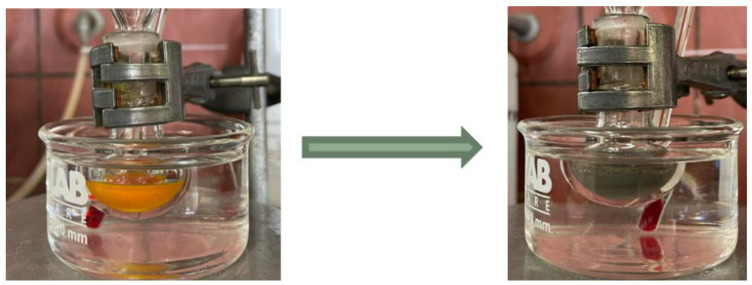
Reaction color before (**left**) and after (**right**) the addition of the palladium catalyst.

It is important to note that although several glycerol-containing NaDES have been recently applied as solvents for Suzuki–Miyaura cross coupling [17,18], the role of glycerol in the reaction outcome is still under investigation. In this context, during the optimization study of the model reaction of the present work, when the Choline chloride/Glycerol = 1:2 and Betaine/Glycerol = 1:2 NaDES were used, it was observed that after the addition of the palladium catalyst, the color of the reaction mixture turned darker until a homogenous dark grey color was achieved in less than 5 min, indicating the formation of Pd nanoparticles (PdNPs) (Figure 1).

The synthesis of PdNPs in deep eutectic solvents has only recently been reported in the literature, and it is usually performed via techniques such as electrodeposition [28]. In the work of Leal-Duaso et al., in 2021, the synthesis of PdNPs in glycerol-based DES by heating at 80 °C was reported, and the reduction of Pd(II) to Pd(0) was attributed to the small amounts of water present in the DES [29]. The formation of PdNPs in polyol-containing solvents has been attributed to the reducing character of polyols in high temperatures, also known as the “polyol process” [30].

In the extensive mechanistic study reported by Liu et al. in 2020 [31] regarding the synthesis of silver nanoparticles in glycerol, the results suggest that aldehydes (formed by the oxidation of glycerol in the presence of O_2_ dissolved in the reaction mixture) and/or free radicals (formed by the exposure of the mixture to visible light) function as the reducing species.

The reducing mechanism of the choline chloride/urea DES during the formation of gold nanoparticles has been extensively studied by Datta et al. in 2023 [32]. The results showed that the ammonia produced by urea hydrolysis is the reducing agent, whereas choline chloride and its decomposition product (trimethylamine) do not play a role in the reduction process. The stabilization of the gold nanoparticles by the DES was also shown.

In the present work, it can be postulated that the choline chloride/glycerol NADES acts as a reducing agent via free radical formation, taking into account that the reaction mixture is exposed to visible light which promotes the formation of the free radicals. This speculation can be corroborated by the fact that the NADES was successfully recycled and reused three times, and the ^1^H NMR obtained each time did not show any traces of an aldehyde proton. Choline chloride and betaine probably play the role of the stabilizer of the palladium nanoparticles, along with glycerol. However, as we did not perform any mechanistic studies, we should emphasize that these are only speculations based on the literature mentioned above.

In order to further investigate the reducing activity of the three glycerol-based NaDES and pure glycerol, 0.016 mmol (3.8 mg) of Pd(OAc)_2_ was added to 3.1 g of the solvent, and the reaction mixture was heated at 90 °C. After 5 min, a sample was withdrawn from the mixture, and the formation of PdNPs was examined using Dynamic Light Scattering (DLS).

### 2.2. Dynamic Light Scattering (DLS) of the PdNPs

The hypothesis of the synthesis of the palladium nanoparticles in the NaDES was assessed using DLS for the determination of the average hydrodynamic diameter and the polydispersity index of the aqueous dispersion of the PdNPs. Moreover, the zeta potential was measured via electrophoretic light scattering. The results for Choline Chloride/Glycerol = 1:2, Betaine/Glycerol = 1:2 and L-proline/Glycerol = 1:2 NaDES, as well as pure glycerol, are displayed in Table 2.

According to the DLS results, nanoparticles of 128.4 nm and 140.6 nm are formed in the ChCl/Gly = 1:2 and Bet/Gly = 1:2 NaDES, respectively. The PdNPs formed in the ChCl/Gly = 1:2 NaDES present the lowest PDI value (0.343), indicating the most homogeneous dispersion, while the ζ-potential values indicate a satisfactory stability of the aqueous dispersion of the PdNPs in both NaDES (−18.6 and −22.7mV, respectively). In the research work of Garg et al. [33], PdNPs were prepared in glycerol using choline-based ionic liquids as stabilizers at 80 °C for 18 h. However, the researchers reported that the use of Pd(OAc)_2_ as a metal precursor led to the particles’ aggregation, as opposed to our work.

In the Pro/Gly = 1:2 NaDES, nanoparticles with an average hydrodynamic diameter of 467.1 nm and a PDI value of 0.634 are formed, which indicates a heterogenous dispersion and the presence of aggregates in the mixture. In the dispersion of PdNPs formed in pure glycerol, it was observed that very few particles in the nanoscale were formed (25%). It is important to note that the DLS measurements of the PdNPs in the Pro/Gly = 1:2 NaDES as well as in pure glycerol remained practically unchanged even after 5 h of stirring at 90 °C.

Interestingly, the average hydrodynamic diameter of the formed PdNPs seems to have a direct impact on the yield of the desired product **4b** obtained from the model reaction. Specifically, the Suzuki–Miyaura coupling proceeded in high yields (70–95%) when ChCl/Gly = 1:2 and Bet/Gly = 1:2 NaDES were used, in which PdNPs with an average hydrodynamic diameter of 140 and 128 nm, respectively, are formed. On the contrary, in Pro/Gly = 1:2 NaDES (size of PdNPs 467.1 nm), as well as in pure glycerol, in which the majority of the Pd particles are found on a microscale, only traces of the final product were obtained.

### 2.3. TEM Analysis of the PdNPs

**Figure 2:**
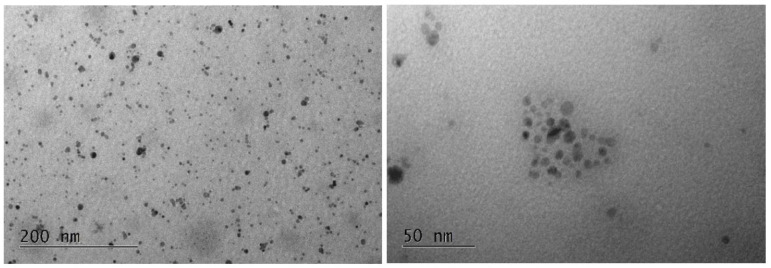
TEM images of the PdNPs.

The morphology of the PdNPs that are formed in the Choline chloride/Glycerol = 1:2 NaDES (0.016 mmol Pd(OAc)_2_, 3.1 g solvent, t = 5 min, 90 °C) was assessed using TEM microscopy (Figure 2).

The TEM images reveal the formation of nanoparticles of a spherical shape with a diameter of 5–10 nm and very few aggregates. NaDES are well known for their stabilizing effect, which is achieved via electrostatic interactions [34,35].

### 2.4. Deacetylation Reaction

During the optimization of the Suzuki–Miyaura reaction for the synthesis of the novel coumarin analogs, a concomitant removal of the acetyl group of compound **3b** was observed in pure glycerol and in the glycerol-containing NaDES, leading to the synthesis of the deacetylated compound **4b**. The presence of hydroxyl groups on the coumarin scaffold is essential for many bioactivities such as antioxidant or cytotoxic activity, and thus, it is important to develop a methodology that provides these compounds directly [24].

**Scheme 2:**
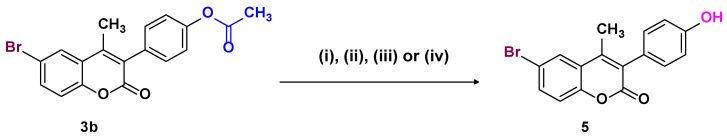
Different conditions tested for the deacetylation reaction: (i) ChCl/Gly = 1:2, K_2_CO_3_, conventional heating (90 °C), (ii) ChCl/Gly = 1:2, K_2_CO_3_, ultrasound irradiation, (iii) Glycerol, K_2_CO_3_, conventional heating (90 °C), (iv) Glycerol, K_2_CO_3_, ultrasound irradiation.

As a result, the appropriate conditions for the deacetylation reaction were examined separately using 3-(4-acetyloxyphenyl)-6-bromo-4-methyl-coumarin **3b** as the substrate, pure glycerol or Choline chloride/Glycerol (ChCl/Gly) = 1:2 NaDES as the solvent and K_2_CO_3_ as the base (Scheme 2). Conventional heating as well as ultrasound irradiation were tested (Table 3).

An effective removal of the acetyl group occurs in both pure glycerol and the Choline chloride/Glycerol = 1:2 NaDES in the presence of potassium carbonate as the base. When conventional heating at 90 °C is applied, the reaction is completed in 3 h with high yields (72–79%) for both solvents, while it is noteworthy that ultrasonic radiation leads to significantly shorter reaction times, 15 min in the NaDES (73% yield) and only 6 min in glycerol with a very high yield (92%). It is important to note that the product is obtained in high purity without requiring further purification in all cases. Thus, the developed synthetic procedure consists of a significantly greener approach than at least one of the widely used conventional deacetylation methods for coumarin derivatives, which involves the use of hydrazine monohydrate in methanol [24,25].

### 2.5. Recyclability and Reusability of the NaDES

**Figure 3:**
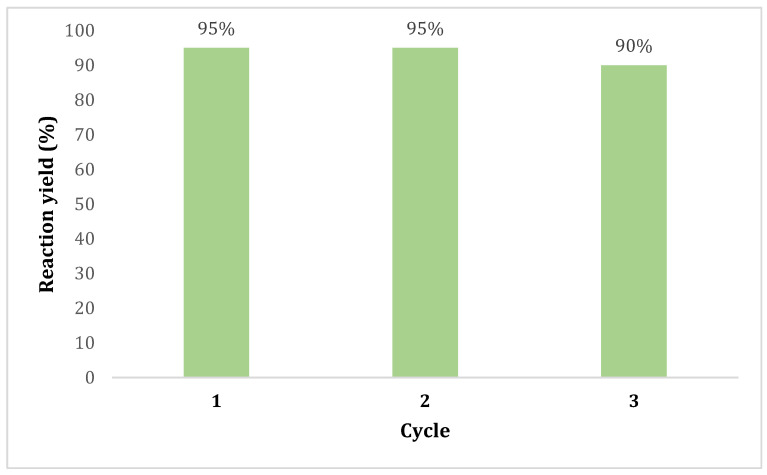
Reuse of Choline chloride/Glycerol = 1:2 NaDES in the Suzuki–Miyaura reaction.

Deep eutectic solvents can be successfully recycled and reused multiple times after their application due to their non-volatile nature, which renders the process more eco-friendly and less cost effective [36]. In this context, the recyclability and reusability of Choline chloride/Glycerol = 1:2 NaDES in the Suzuki–Miyaura cross coupling reaction were examined (Figure 3). The NaDES that was applied to the model reaction under the optimized conditions was recovered after the aqueous work-up of the reaction by removing the water under vacuum (see the Experimental Section). After establishing the purity of the NaDES using ^1^H NMR spectroscopy, it was then used without any further purification for another cycle.

Following this process, Choline chloride/Glycerol = 1:2 NaDES was successfully recycled and reused twice without any significant decrease in the reaction yield, indicating the potential of the solvent for facile recycling and reuse and thus rendering this synthetic approach even more sustainable.

### 2.6. Broadening the Scope of the Reaction

To highlight the overall potential of the proposed synthetic protocol as well as in order to obtain multi-substituted coumarin analogs with potential cytotoxic activity, different substituted aryl or heteroaryl boronic acids were used in the Suzuki–Miyaura cross-coupling reaction under the optimized conditions.

After establishing that the deacetylation reaction takes place in the conditions that were defined as optimum for the Suzuki–Miyaura coupling reaction (Choline chloride/Glycerol = 1:2 NaDES, K_2_CO_3_ and Pd(OAc)_2_, 90 °C, 24 h), we performed the coupling reactions in two ways: Route A involved adding all the reactants in the NaDES from the start and Route B involved performing first the deacetylation of coumarin and then the addition of the catalyst and the boronic acid. We observed that, when the second route was followed, the reaction yield and the product purity were significantly higher; thus, we proceeded to expand the scope of the reaction using Route B. Of course, in the case of coumarin **3a**, which does not possess an acetyloxy group, Route A was followed.

**Scheme 3:**
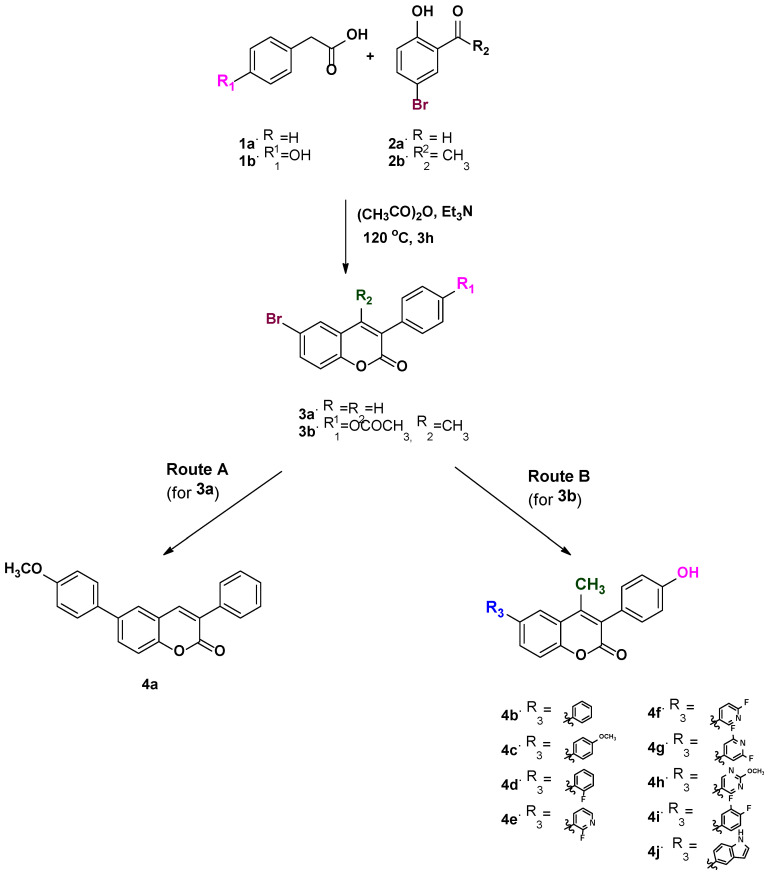
Conditions for Route A: **3a**, R_3_-B(OH)_2_, Choline chloride/Glycerol = 1:2, K_2_CO_3_, Pd(OAc)_2_ (0.025 mol/mol Pd(II)/**3a**), 90 °C, 24 h; Conditions for Route B: i. **3b**, Choline chloride/Glycerol = 1:2, K_2_CO_3_, 90 °C, 3h, ii. R_3_-B(OH)_2_, Pd(OAc)_2_ (0.025 mol/mol Pd(II)/**3b**), 90 °C, 21 h.

As a result, ten novel 6-subsituted-3-aryl-coumarin derivatives (**4a**–**4j**) were synthesized, as shown in Scheme 3.

The novel coumarin analogs were obtained in good to excellent yields (50–97%) and high purity. The structures of the products were determined via ^1^H and ^13^C NMR spectroscopy as well as high-resolution mass spectrometry (HR-MS).

**Figure 4:**
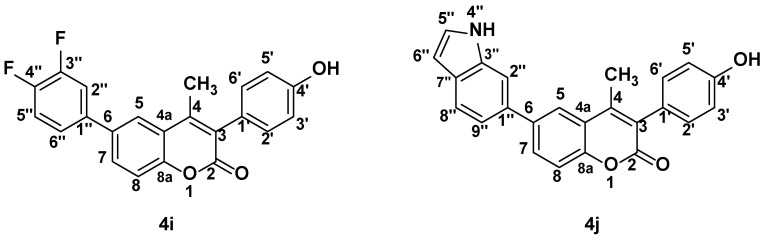
Chemical structure of compounds **4i** and **4j**.

2D ^1^H-^1^H COSY and NOESY, 2D ^1^H-^13^C HSQC and HMBC as well as H2BC NMR experiments were implemented for the complete elucidation of the chemical structure of compounds **4i** and **4j**, which were selected as representative compounds (Figure 4). The 2D NMR spectra of compounds **4i** and **4j** are available in the Supplementary Materials.

The assignment of the ^1^H resonance peaks of compound **4i** has been enabled via the COSY correlations observed between H3′/H5′ and H2′/H6′, between the coumarin skeleton protons H7-H8 and the long range H7-H5 and between H5″-H6″ and the long range H6″-H2″. NOESY spectroscopy verified the COSY correlations and further revealed NOE interaction between the methyl group and H5 as well as between H6″ and the coumarin protons H7 and H5. The connectivity of each proton with its respective carbon was identified through the HSQC experiment, also probing to ^13^C-^19^F couplings for the atoms in the aryl substituent at the position-6 of the coumarin scaffold. Finally, a complete elucidation of the chemical structure of compound **4i** was achieved using the heteronuclear HMBC and H2BC experiments, which allowed for the detailed assignment of all carbon resonances (Table 4). The key interactions, which were observed in the HMBC spectrum, are presented in Figure 4.

**Figure 5:**
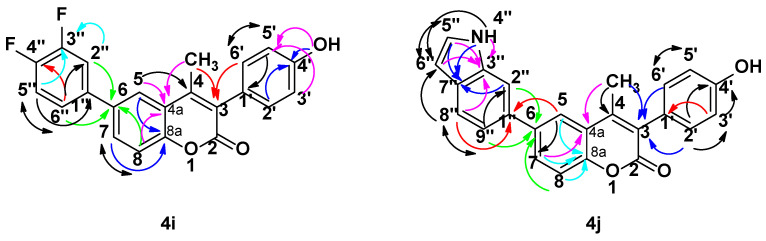
Main HMBC and H2BC correlations of compounds **4i** and **4j**.

Regarding compound **4j**, ^1^H resonances of the indole moiety were unambiguously assigned via the observed COSY correlations between H5″-H6″, H8″-H9″, a long COSY correlation between H2″-H9″, the COSY correlation between NH with H5″ and its long COSY interaction with H6″. Moreover, similar to compound **4i**, COSY correlations were monitored between the coumarin skeleton protons H7 and H8 as well as between the 3-aryl moiety protons H3′/H5′ and H2′/H6′. Furthermore, the observed NOE interactions between the methyl group at position 4 with H2′/H6′ and H5 as well as between NH with the indole protons H2″ and H5″ were in line with the proposed assignment. The connectivity of each proton with its respective carbon was identified through the HSQC experiment, while the experiment also elucidated the overlapping ^1^H resonances of H5″ and H9″. In continuation, HMBC spectroscopy was used in order to verify our assignment hypothesis, and the main interactions which led to the detailed assignment of the protons and carbons of **4j** are presented in Figure 5. For instance, HMBC enabled the identification of the overlapping C4a and C8″ carbon resonances as well as those of C3 and C5″ and further confirmed the overlapping ^1^H signals of H5″ and H9″ of the indole ring. Lastly, H2BC signals further validated the proposed assignment.

### 2.7. Cytotoxic Activity

The cytotoxic activity of the novel coumarin derivatives at a concentration of 100 μΜ was tested against the human squamous cancer cell line, A431, and the results are displayed in Table 5.

Coumarin analog **5**, which was used as the substrate for the preparation of the 6-aryl-coumarin derivatives via the Suzuki–Miyaura coupling reaction, exhibited 58.1% cytotoxic activity against the A431 cancer line at the concentration of 100 µM. The most potent cytotoxic agents were compounds **4i** (67.9%) and **4g** (69.9%), bearing two fluorine groups on the aryl or pyridine moiety of position 6, respectively. This observation is in accordance with our previous work in which several fluorinated coumarin analogs exhibited significant cytotoxic activity against the A549 (adenocarcinomic human alveolar basal epithelial cells) and A375 (human melanoma) cancer cell lines [24]. However, the position and the number of the fluorine substituents significantly affects the activity. Specifically, coumarin analog **4e**, which bears only one fluorine group at position 2″ of the pyridine moiety, exhibited low cytotoxicity (36.4%), while compound **4d**, which bears a 4″-fluoro-aryl substituent, was found to be inactive. Furthermore, compound **4b** exhibited significant activity (57.9%), while the insertion of a methoxy group at the para position of the aryl group of position 6 (**4c**), led to the complete loss of activity.

## 3. Materials and Methods

### 3.1. Chemicals and Instruments

The chemicals used for the synthesis of the novel compounds were purchased from Sigma-Aldrich (Burlington, MA, USA), Fluka (Buchs, Switzerland), Alfa-Aesar (Lancashire, UK) and Acros (Fukuoka, Japan) and were used without further purification. The reactions were monitored by TLC (Macherey-Nagel, Düren, Germany, 0.20 mm layer thickness plates). Flash column chromatography purifications were performed over silica gel (Panreac, Darmstadt, Germany, 40–60 microns). NMR spectra were recorded on Varian 300 & 600 MHz spectrometers, and the HR-MS spectra were recorded on a UHPLC-MSn Orbitrap Velos-Thermo mass spectrometer (Thermo Scientific, Waltham, Massachusetts, U.S.) (both instruments located at the Institute of Chemical Biology, National Hellenic Research Foundation). For the cell-based studies, Dulbecco’s phosphate-buffered saline (DPBS), without CaCl_2_ and MgCl_2_, pH 7.4, dimethyl sulfoxide (DMSO) and 3-(4,5-dimethylthiazol-2-yl)-2,5-diphenyl-2*H*-tetrazolium bromide (MTT) were obtained from Merck Millipore (Billerica, MA, USA). Fetal Bovine Serum, FBS, Dulbecco’s Modified Eagle Medium high glucose, L-Glutamine and antibiotic–antimitotic were purchased from Gibco (Seoul, Republic of Korea).

### 3.2. General Procedure for the NADES Preparation

The NaDES were prepared using the heating and stirring method with slight modifications [37]. For the glycerol-based NaDES (ChCl/Gly, Bet/Gly, Pro/Gly), the hydrogen bond donor and glycerol were mixed in the appropriate molar ratio (1:2) in a round-bottomed flask and stirred in an oil bath with a magnetic stirrer at 60 °C until a clear liquid was formed. For the monosaccharide-containing NaDES, fructose or glucose, urea and water were mixed in the appropriate molar ratio (1:1.5:1) and stirred in an oil bath with a magnetic stirrer at 90 °C until a clear viscous liquid was formed.

### 3.3. General Procedure for the Synthesis of 3-Aryl-6-Bromo-Coumarin Analogs (***3a***–***3b***)

The 3-aryl-6-bromo-coumarin analogs **3a**–**3b** were synthesized using the synthetic procedure reported in our previous work [24]. A mixture of the appropriate phenylacetic acid (1 eq) and appropriately substituted acetophenone or benzaldehyde (1.05 eq) in the presence of triethylamine (3.1 eq) in acetic anhydride was refluxed for 3 hr. After the completion of the reaction, water was added and the mixture was extracted with dichloromethane. The organic phase was separated, dried over anhydrous Na_2_SO_4_, filtered and concentrated in vacuo to give the crude products. The products were triturated with methanol.

*6-bromo-3-phenyl-2H-chromen-2-one* (**3a**). Based on the general procedure, phenylacetic acid (500.0 mg, 3.67 mmol) and 5-bromo-2-hydroxybenzaldehyde (775.0 mg, 3.86 mmol) are dissolved in 3.3 mL of acetic anhydride and 1.6 mL of triethylamine. A light brown solid is obtained (917.9 mg, 83% yield). Melting point 176–176 °C **^1^H NMR** (600 MHz, CDCl_3_): δ (ppm) 7.72 (s, 1H), 7.69 (d, *J* = 7.2 Hz, 3H), 7.61 (dd, *J* = 9 Hz, *J* = 2.4 Hz, 1H), 7.47–7.42 (m, 3H), 7.25 (d, *J* = 9 Hz, 1H).

*3-(4-acetyloxyphenyl)-6-bromo-4-methyl-2H-chromen-2-one* (**3b**). Based on the general procedure, 4-hydroxy-phenylacetic acid (600.7 mg, 3.95 mmol) and 5-bromo-2-hydroxyacetophenone (891.4 mg, 4.15 mmol) are dissolved in 4.4 mL of acetic anhydride and 1.7 mL of triethylamine, under a nitrogen atmosphere. A yellow solid is obtained (802.1 mg, 54.4% yield). Melting point 186–187 °C, **^1^H NMR** (600 MHz, CDCl_3_): δ (ppm) 7.79 (d, *J* = 2.4 Hz, 1H), 7.63 (dd, *J* = 9 Hz, *J* = 2.4 Hz, 1H), 7.31 (d, *J* = 8.4 Hz, 2H), 7.26 (d, *J* = 9 Hz, 1H), 7.20 (d, *J* = 8.4 Hz, 2H), 2.32 (s, 3H), 2.32 (s, 3H).

### 3.4. General Procedure for the of Synthesis of 3,6-Disubstituted Coumarin Analogs

*Route A: Synthetic procedure for 6-(4-methoxyphenyl)-3-phenyl-2H-chromen-2-one (* **4a** *).*

In an oven-dried round bottomed flask, Choline chloride/Glycerol = 1:2 NaDES is added and heated at 90 °C. Then, 6-bromo-3-phenylcoumarin (**3a**) (102.8 mg, 0.34 mmol), 4-methoxyphenylboronic acid (57.1 mg, 0.38 mmol), K_2_CO_3_ (59.4 mg, 0.43 mmol) and a catalytic amount of Pd(OAc)_2_ (1.9 mg, 0.0085 mmol) are added in 1.7 g NaDES, and the mixture is heated at 90 °C for 24 h under an inert atmosphere. After the reaction is completed, the mixture is quenched with cold water and the aqueous phase is extracted with ethyl acetate. The organic layer is then dried over anhydrous Na_2_SO_4_ and evaporated. After flash column chromatography (petroleum ether/ethyl acetate = 4:1), the product was obtained as a yellow powder (69.9 mg, 56% yield). Melting point >250 °C (decomp), **^1^H NMR** (300 MHz, CDCl_3_): δ (ppm) 7.87 (s, 1H), 7.74–7.68 (m, 5H), 7.53 (d, *J* = 8.7 Hz, 2H), 7.47–7.40 (m, 3H), 7.01 (d, *J* = 8.7 Hz, 2H), 3.87 (s, 3H, OCH_3_), **^13^C NMR** (75 MHz, CDCl_3_): δ (ppm) 160.7, 159.6, 152.6, 140.1, 137.6, 134.8, 132.1, 131.0, 130.1, 129.0, 128.6, 128.2, 125.6, 120,0, 116.8, 114.6, 55.5, **HRMS** calcd for C_22_H_17_O_3_ (M + H)^+^: *m*/*z*: 329.1099, found: 329.1171.

*Route B: General procedure for the synthesis of 3,6-disubstituted coumarin analogs* **4b**–**4j**

In an oven-dried round bottomed flask, Choline chloride/Glycerol = 1:2 NaDES is added and heated at 90 °C. Then, 3-(4-acetyloxyphenyl)-6-bromo-4-methyl-coumarin (**3b**) (1 eq) and K_2_CO_3_ (1.25 eq) are added and the mixture is stirred at 90 °C for 3 h under an inert atmosphere so that the deacetylation reaction is completed, as confirmed by TLC. After the completion of the deacetylation reaction, the palladium (II) catalyst (Pd(OAc)_2_) is added (0.025 eq) and the mixture is stirred at 90 °C for 21 h. After the reaction is completed, the mixture is quenched with cold water and the aqueous phase is extracted with ethyl acetate. The organic layer is then dried over anhydrous Na_2_SO_4_ and evaporated. The purification of the final product, when needed, is performed via flash column chromatography in a mixture of petroleum ether or hexane and ethyl acetate in an appropriate ratio.

*3-(4-hydroxyphenyl)-4-methyl-6-phenyl-2H-chromen-2-one* (**4b**). The compound was synthesized according to the general procedure (route B), starting from 3-(4-acetyloxyphenyl)-6-bromo-coumarin (**3b**) (111.1 mg, 0.30 mmol), phenylboronic acid (39.9 mg, 0.33 mmol), K_2_CO_3_ (51.1 mg, 0.37 mmol) and a catalytic amount of Pd(OAc)_2_ (1.7 mg, 0.0075 mmol) in 1.5 g NaDES. After flash column chromatography on silica gel (petroleum ether/ethyl acetate = 4:1), the product was obtained as an off-white powder (103.0 mg, 95% yield). Melting point >250 °C (decomp), **^1^H NMR** (300 MHz, DMSO-d_6_): δ (ppm) 7.79 (s, 1H), 7.70 (d, *J* = 8.4 Hz, 1H), 7.58 (d, *J* = 7.5 Hz, 2H), 7.45–7.38 (m, 4H), 7.13 (d, *J* = 7.2 Hz, 2H), 6.92 (d, *J* = 7.8 Hz, 2H), 2.36 (s, 3H, CH_3_), **^13^C NMR** (75 MHz, DMSO-d_6_): δ (ppm) 160.0, 157.3, 157.2, 151.4, 147.4, 139.1, 136.4, 131.4, 129.7, 129.0, 127.6, 126.9, 124.8, 123.7, 120.6, 118.4, 116.7, 114.9, **HRMS** calcd for C_22_H_17_O_3_ (M + H)^+^: *m*/*z*: 329.1099, found: 329.1171, **HRMS** calcd for C_22_H_15_O_3_ (M − H)^−^: *m*/*z*: 327.1099, found: 327.1019.

*3-(4-hydroxyphenyl)-6-(4-methoxyphenyl)-4-methyl-2H-chromen-2-one* (**4c**). The compound was synthesized according to the general procedure (route B), starting from 3-(4-acetyloxyphenyl)-6-bromo-coumarin (**3b**) (230.0 mg, 0.62 mmol), 4-methoxyphenylboronic acid (103.0 mg, 0.68 mmol), K_2_CO_3_ (106.4 mg, 0.77 mmol) and a catalytic amount of Pd(OAc)_2_ (3.6 mg, 0.016 mmol) in 3.1 g NaDES. The product was obtained as a white powder (222.2 mg, 66% yield). Melting point >250 °C (decomp), **^1^H NMR** (300 MHz, DMSO-d_6_): δ (ppm) 9.62 (s, 1H, OH), 8.01 (d, *J* = 2.1 Hz, 1H), 7.91 (dd, *J* = 8.7 Hz, *J* = 2.1 Hz, 1H), 7.78 (d, *J* = 7.2 Hz, 2H), 7.53–7.48 (m, 3H), 7.40 (t, *J* = 7.8 Hz, 1H), 7.15 (d, *J* = 8.7 Hz, 2H), 6.85 (d, *J* = 8.7 Hz, 2H), 3.81 (s, 3H, OCH_3_), 2.38 (s, 3H, CH_3_), **^13^C NMR** (75 MHz, DMSO-d_6_): δ (ppm) 160.0, 157.1, 151.4, 147.4, 139.1, 136.4 131.4, 129.8, 129.0, 127.7, 126.9, 126.6, 124.8, 123.7, 120.6, 116.7, 114.9, 16.6, **HRMS** calcd for C_23_H_17_O_4_ (M − H)^−^: *m*/*z*: 357.1205, found: 357.1126.

*6-(2-fluorophenyl)-3-(4-hydroxyphenyl)-4-methyl-2H-chromen-2-one (***4d***).* The compound was synthesized according to the general procedure (route B), starting from 3-(4-acetyloxyphenyl)-6-bromo-coumarin (**3b**) (41.4 mg, 0.13 mmol), 2-fluorophenylboronic acid (19.2 mg, 0.14 mmol), K_2_CO_3_ (22.1 mg, 0.16 mmol) and a catalytic amount of Pd(OAc)_2_ (0.7 mg, 0.0033 mmol) in 0.65 g NaDES. The product was obtained as a light yellow powder (34.0 mg, 71% yield). Melting point 253–254 °C, **^1^H NMR** (300 MHz, DMSO-d_6_): δ (ppm) 9.63 (br, 1H), 7.93 (s, 1H), 7.79 (dt, *J* = 8.7 Hz, *J* = 1.8 Hz, 1H), 7.65 (td, *J* = 8.4 Hz, *J* = 1.8 Hz, 1H), 7.52 (d, *J* = 8.4 Hz, 1H), 7.47–7.43 (m, 1H), 7.38–7.32 (m, 2H), 7.15 (d, *J* = 8.4 Hz, 2H), 6.85 (d, *J* = 8.4 Hz, 2H), 2.33 (s, 3H, CH_3_), **^13^C NMR** (75 MHz, DMSO-d_6_): δ (ppm) 160.0, 159.1 (d, *J* = 244.6 Hz), 157.2, 151.5, 147.2, 131.8 (d, *J* = 3.0 Hz), 131.4, 131.2, 131.0 (d, *J* = 3.1 Hz), 129.9 (d, *J* = 8.3 Hz), 127.2 (d, *J* = 13.0 Hz), 126.7, 126.0 (d, *J* = 2.4 Hz), 125.1 (d, *J* = 3.5 Hz), 124.8, 120.5, 116.4, 116.2 (d, *J* = 22.2 Hz), 114.9, 16.5, **HRMS** calcd for C_22_H_14_O_3_F (M − H)^−^: *m*/*z*: 345.3511, found: 345.0933.

*6-(2-fluoropyridin-3-yl)-3-(4-hydroxyphenyl)-4-methyl-2H-chromen-2-one* (**4e**). The compound was synthesized according to the general procedure (route B), starting from 3-(4-acetyloxyphenyl)-6-bromo-coumarin (**3b**) (100.0 mg, 0.27 mmol), 2-fluoropyridin-3yl-boronic acid (41.5 mg, 0.29 mmol), K_2_CO_3_ (45.6 mg, 0.33 mmol) and a catalytic amount of Pd(OAc)_2_ (1.5 mg, 0.0068 mmol) in 1.4 g NaDES. The product was obtained as a light yellow powder (91.4 mg, 95% yield). Melting point >250 °C (decomp), **^1^H NMR** (300 MHz, DMSO-d_6_): δ (ppm) 9.63 (br, 1H), 8.28–8.21 (m, 2H), 8.01 (s, 1H), 7.86 (d, *J* = 8.7 Hz, 1H), 7.54 (d, *J* = 8.4 Hz, 1H), 7.52–7.49 (m, 1H), 7.15 (d, *J* = 8.4 Hz, 2H), 6.85 (d, *J* = 8.4 Hz, 2H), 2.34 (s, 3H, CH_3_), **^13^C NMR** (75 MHz, DMSO-d_6_): δ (ppm) 159.9, 159.6 (d, *J* = 235.4 Hz), 157.2, 151.8, 147.1, 146.7 (d, *J* = 14.9 Hz), 141.7 (d, *J* = 4.0 Hz), 131.7 (d, *J* = 3.2 Hz), 131.4, 129.6 (d, *J* = 5.1 Hz), 126.8, 126.2, 124.7, 122.7 (d, *J* = 4.2 Hz), 122.0 (d, *J* = 28.2 Hz), 120.6, 116.6, 114.9, 30.7, **HRMS** calcd for C_21_H_13_O_3_NF (M − H)^−^: *m*/*z*: 348.0958, found: 348.0882.

*6-(4-fluoropyridin-3-yl)-3-(4-hydroxyphenyl)-4-methyl-2H-chromen-2-one* (**4f**). The compound was synthesized according to the general procedure, starting from 3-(4-acetyloxyphenyl)-6-bromo-coumarin (**3b**) (100.0 mg, 0.27 mmol), 4-fluoropyridin-3-ylboronic acid (41.5 mg, 0.29 mmol), K_2_CO_3_ (45.6 mg, 0.33 mmol) and a catalytic amount of Pd(OAc)_2_ (1.5 mg, 0.0068 mmol) in 1.4 g NaDES. After flash column chromatography on silica gel (petroleum ether/ethyl acetate = 7:3), the product was obtained as a dark yellow powder (63.7 mg, 68% yield). Melting point >250 °C (decomp), **^1^H NMR** (300 MHz, DMSO-d_6_): δ (ppm) 9.64 (br, 1H), 8.67 (s, 1H), 8.40 (td, *J* = 7.8 Hz, *J* = 1.8 Hz, 1H), 8.07 (s, 1H), 7.95 (d, *J* = 8.4 Hz, 1H), 7.52 (d, *J* = 8.7 Hz, 1H), 7.31 (dd, *J* = 8.7 Hz, *J* = 2.4 Hz, 1H), 7.14 (d, *J* = 8.4 Hz, 2H), 6.85 (d, *J* = 8.4 Hz, 2H), 2.38 (s, 3H, CH_3_), **^13^C NMR** (75 MHz, DMSO-d_6_): δ (ppm) 162.3 (d, *J* = 235.4 Hz), 160.0, 157.2, 151.8, 147.4, 145.7 (d, *J* = 15.2 Hz), 140.7 (d, *J* = 8.0 Hz), 133.3 (d, *J* = 4.4 Hz), 132.1, 131.4, 129.9, 126.8, 124.7, 124.2, 120.8, 117.0, 114.9, 109.7 (d, *J* = 37.5 Hz), 16.6, **HRMS** calcd for C_21_H_15_O_3_NF (M + H)^+^: *m*/*z*: 348.0958, found: 348.1030.

*6-(2,4-difluoropyridin-3-yl)-3-(4-hydroxyphenyl)-4-methyl-2H-chromen-2-one* (**4g**). The compound was synthesized according to the general procedure (route B), starting from 3-(4-acetyloxyphenyl)-6-bromo-coumarin (**3b**) (100.6 mg, 0.27 mmol), 2,4-difluoropyridin-3-ylboronic acid (47.1 mg, 0.30 mmol), K_2_CO_3_ (47.0 mg, 0.34 mmol) and a catalytic amount of Pd(OAc)_2_ (1.5 mg, 0.0068 mmol) in 1.4 g NaDES. After flash column chromatography (hexane/ethyl acetate = 3:1), the product was obtained as a light yellow powder (49.3 mg, 50% yield). Melting point >260 °C (decomp), **^1^H NMR** (300 MHz, DMSO-d_6_): δ (ppm) 9.64 (br, 1H), 8.45 (d, *J* = 8.1 Hz, 1H), 8.01 (s, 1H), 7.84 (d, *J* = 7.2 Hz, 1H), 7.56 (d, *J* = 8.7 Hz, 1H), 7.35 (d, *J* = 7.2 Hz, 1H), 7.15 (d, *J* = 8.1 Hz, 2H), 6.85 (d, *J* = 8.1 Hz, 2H), 2.34 (s, 3H, CH_3_), **HRMS** calcd for C_21_H_12_F_2_NO_3_ (M − H)^−^: *m*/*z*: 364.0863, found: 364.0783.

*3-(4-hydroxyphenyl)-6-(2-methoxypyrimidin-5-yl)-4-methyl-2H-chromen-2-one* (**4h**). The compound was synthesized according to the general procedure (route B), starting from 6-bromo-3-(4-hydroxyphenyl)-coumarin (**3b**) (81.0 mg, 0.25 mmol), 2-methoxypyrimidine-5-boronic acid (41.8 mg, 0.27 mmol), K_2_CO_3_ (42.8 mg, 0.31 mmol) and a catalytic amount of Pd(OAc)_2_ (1.4 mg, 0.0063 mmol) in 1.3 g NaDES. The product was obtained as a dark yellow powder (56.7 mg, 63% yield). Melting point >250 °C (decomp), **^1^H NMR** (300 MHz, DMSO-d_6_): δ (ppm) 9.62 (br, 1H), 9.05 (s, 2H), 8.10 (d, *J* = 2.1 Hz, 1H), 7.97 (dd, *J* = 8.7 Hz, *J* = 2.1 Hz, 1H), 7.54 (d, *J* = 8.7 Hz, 1H), 7.14 (d, *J* = 8.7 Hz, 2H), 6.85 (d, *J* = 8.7 Hz, 2H), 3.98 (s, 3H, OCH_3_), 2.38 (s, 3H, CH_3_), **^13^C NMR** (150 MHz, DMSO-d_6_): δ (ppm) 164.9, 160.5, 157.8, 157.4, 152.0, 147.9, 131.8, 130.5, 129.8, 127.0, 126.9, 125.1, 123.9, 121.2, 117.4, 115.3, 55.2, 16.9, **HRMS** calcd for C_21_H_15_O_4_N_2_ (M − H)^−^: *m*/*z*: 359.3627, found: 359.1049.

*6-(3,4-difluorophenyl)-3-(4-hydroxyphenyl)-4-methyl-2H-chromen-2-one* (**4i**). The compound was synthesized according to the general procedure (route B), starting from 6-bromo-3-(4-hydroxyphenyl)-coumarin (**3b**) (100.0 mg, 0.30 mmol), 3,4-difluorophenylboronic acid (52.1 mg, 0.33 mmol), K_2_CO_3_ (52.4 mg, 0.38 mmol) and a catalytic amount of Pd(OAc)_2_ (1.7 mg, 0.0075 mmol) in 1.5 g NaDES. The product was obtained as a brown powder (65.6 mg, 60% yield). Melting point >250 °C (decomp), **^1^H NMR** (600 MHz, DMSO-d_6_): δ (ppm) 9.64 (br, 1H, OH), 8.03 (d, *J* = 1.8 Hz, H5), 7.95 (dd, *J* = 7.8 Hz, *J* = 1.8 Hz, 1H, H2″), 7.93 (dd, *J* = 9.0 Hz, *J* = 1.8 Hz, 1H, H7), 7.65 (br, 1H, H6″), 7.55 (q, *J* = 8.4 Hz, 1H, H5″), 7.50 (d, *J* = 9.0 Hz, 1H, H8), 7.13 (d, *J* = 7.2 Hz, 2H, H2′/H6′), 6.84 (d, *J* = 7.2 Hz, 2H, H3′/H5′), 2.38 (s, 3H, CH_3_), **^13^C NMR** (150 MHz, DMSO-d_6_): δ (ppm) 160.1. 157.3, 151.8, 149.9 (dd, *J* = 244.1 Hz, *J* = 12.6 Hz), 149.3 (dd, *J* = 245.0 Hz, *J* = 12.6 Hz), 147.6, 136.7, 134.3, 131.6, 129.9, 126.8, 124.9, 124.1, 123.8 (dd, *J* = 6.5 Hz, *J* = 3.0 Hz), 120.7, 118.1 (d, *J* = 17.0 Hz), 116.9, 116.2 (dd, *J* = 17.7 Hz), 115.02, 16.7, **HRMS** calcd for C_22_H_13_O_3_F_2_ (M − H)^−^: *m*/*z*: 363.0911, found: 363.0832.

*3-(4-hydroxyphenyl)-6-(1H-indol-6-yl)-4-methyl-2H-chromen-2-one* (**4j**). The compound was synthesized according to the general procedure (route B), starting from 6-bromo-3-(4-hydroxyphenyl)-coumarin (**3b**) (60.0 mg, 0.18 mmol), 1H-indol-6-ylboronic acid (36.5 mg, 0.29 mmol), K_2_CO_3_ (31.8 mg, 0.23 mmol) and a catalytic amount of Pd(OAc)_2_ (1.0 mg, 0.0045 mmol) in 0.9 g NaDES. The product was obtained as a brown powder (45.8 mg, 69% yield). Melting point >250 °C (decomp), **^1^H NMR** (600 MHz, DMSO-d_6_): δ (ppm), 11.20 (s, 1H, ΝH), 9.62 (s, 1H, OH), 8.00 (d, *J* = 2.1 Hz, 1H, H5), 7.92 (dd, *J* = 8.4 Hz, *J* = 2.1 Hz, 1H, H7), 7.73 (s, 1H, H2″), 7.65 (d, *J* = 8.1 Hz, 1H, H8″), 7.49 (d, *J* = 8.7 Hz, 1H, H8), 7.41–7.39 (m, 2H, H5″& H9″), 7.15 (d, *J* = 8.4 Hz, 2H, H2′/H6′), 6.85 (d, *J* = 8.7 Hz, 2H, H3′/H5′), 6.47 (br, H6″), 2.39 (s, 3H, 1H, CH_3_), **^13^C NMR** (150 MHz, DMSO-d_6_): δ (ppm) 160.1, 157.1, 150.9, 147.5, 138.0, 136.5, 132.3, 131.5, 129.9, 127.3, 126.4, 126.4, 124.9, 123.4, 120.5, 120.5, 118.5, 116.6, 114.9, 109.7, 101.0, 16.6, **HRMS** calcd for C_24_H_16_O_3_N (Μ − 3H)^−^: *m*/*z*: 366.1365, found: 366.1130.

### 3.5. General Procedure for the Synthesis of 6-Bromo-3-(4-Hydroxyphenyl)-4-Methyl-Coumarin (**5**)

#### 3.5.1. Conventional Heating

In a round bottomed flask, 3-(4-acetyloxyphenyl)-6-bromo-4-methyl-coumarin (**3b**) (100.5 mg, 0.27 mmol) is added in 1.4 g solvent (ChCl/Gly = 1:1 or glycerol) along with K_2_CO_3_ (46.5 mg, 0.34 mmol). The mixture is stirred at 90 °C for 3 h and then water is added (4.2 mL), and the precipitate is filtered off by vacuum filtration. The desired product is received in high purity without further purification.

#### 3.5.2. Ultrasound Irradiation

In a glass vessel, 3-(4-acetyloxyphenyl)-6-bromo-4-methyl-coumarin (**3b**) (77.0 mg, 0.21 mmol) is added in 1.1 g solvent (ChCl/Gly = 1:1 or glycerol) along with K_2_CO_3_ (36.6 mg, 0.26 mmol). The mixture is irradiated using an ultrasound probe at 30% amplitude (120 W) until the reaction is completed, as indicated by TLC. After the addition of water (3.3 mL), the precipitate formed is filtered off by vacuum filtration. The product is received in high purity.

*6-bromo-3-(4-hydroxyphenyl)-4-methyl-coumarin* (**5**). The product is obtained as an off-white powder. Melting point >250 °C, **^1^H NMR** (300 MHz, DMSO-*d*_6_): δ (ppm) 9.64 (s, 1H, OH), 7.97 (d, *J* = 2.4 Hz, 1H), 7.77 (dd, *J* = 8.7 Hz, *J* = 2.4 Hz, 1H), 7.39 (d, *J* = 8.7 Hz, 1H), 7.13 (d, *J* = 8.4 Hz, 2H), 6.83 (d, *J* = 8.4 Hz, 2H), 2.27 (s, 3H, CH_3_).

#### 3.5.3. NaDES Recycling

Upon the completion of the model reaction, as described in Section 2.5, water was added to the mixture and the aqueous phase containing the NaDES was extracted with ethyl acetate to isolate the coumarin product. The NaDES was then recovered after the vacuum evaporation of the aqueous phase, its purity was confirmed by ^1^H NMR spectroscopy and it was used for the next reaction.

#### 3.5.4. 2D NMR Spectroscopy

The samples were dissolved in DMSO-d_6_ and the 2D NMR spectra were recorded on a 600 MHz Varian spectrometer equipped with a ^1^H{^13^C,^15^N} cryoprobe. Spectral assignment was facilitated through the use of 2D NMR homo- and heteronuclear experiments (2D ^1^H–^1^H COSY & NOESY and ^1^H–^13^C HSQC, HMBC and H2BC). The homonuclear spectra were acquired with 2k–6k points, 4–32 scans and 256–384 increments in the t1 dimension. A mixing time of 200 ms was applied to the NOESY experiments. The heteronuclear experiments were run with 2–8 scans and 96–200 increments. The experimental data were processed using Openvnmrj 1.1 revA (VARIAN) and Mnova 6.0.2 (MestreLab Research) software. The spectra were referenced through the solvent ^1^H and ^13^C resonance peaks.

#### 3.5.5. High-Resolution Mass Spectrometry

The HR-MS spectra were recorded on a UHPLC-MSn Orbitrap Velos-Thermo mass spectrometer (located at the Institute of Chemical Biology, National Hellenic Research Foundation) using Heated Electrospray Ionization (HESI). Concentrations of 10 ppm of the samples in methanol were prepared. The spectra were acquired using a flow rate of 10 μL/min, source voltage of 4 kV, source temperature of 200 °C, sheath gas flow rate of 10 °C, aux gas flow rate of 5 and capillary temperature of 275 °C. 

### 3.6. General Procedure of the Synthesis and Characterization of the PdNPs

To evaluate the formation of the PdNPs in the glycerol-based NaDES or in pure glycerol, in a round-bottom flask, 3.6 mg (0.016 mmol) of Pd(OAc)_2_ are dispersed in 1.5 g of the solvent, and the mixture is heated at 90 °C, under a nitrogen atmosphere. A sample is withdrawn at 5 min and is appropriately diluted for the Dynamic Light Scattering and Transmission Electron Microscopy experiments.

#### 3.6.1. Determination of Particle Size, Polydispersity Index (PDI) and Zeta Potential

The Malvern Nano ZS zetasizer was used to measure the mean particle size and PDI values of the formed PdNPs via dynamic light scattering (DLS) and the zeta potential values via electrophoretic light scattering. The measurements were recorded at a temperature of 25 °C, after the suitable dilution of the samples in double ionized water (pH 7.0). All experiments were measured in triplicate.

#### 3.6.2. Transmission Electron Microscopy (TEM)

Nanoscale investigation was performed with a high-resolution JEOL (Peabody, MA, USA) JEM-2100 LaB6 transmission electron microscope (HRTEM), operating at 200 kV. An aqueous dispersion of the PdNPs was prepared and was treated with ultrasound to disaggregate the agglomerated particles. Then, a drop of the suspension was placed on a 300-mesh carbon-coated copper grid and air-dried overnight.

### 3.7. Cell Culture Conditions

The human epidermoid cancer cell line A431 was obtained from the American Type Culture Collection (ATCC). The A431 cells were cultivated in 75 cm^2^ culture flasks (Corning, NY, USA) in Earle’s Minimal Essential Medium (MEM), with high glucose, supplemented with 10% heat-inactivated Fetal Bovine Serum, FBS and 0.1% antibiotic–antimitotic. The cells were kept at 37 °C in a 5% CO_2_ humidified incubator, trypsinized and re-seeded into the fresh medium every 3–5 days.

### 3.8. Cell Viability Evaluation

Cell viability was assessed by the MTT [3-(4,5-dimethylthiazol2-yl)-2,5-diphenyl-2H-tetrazolium bromide] colorimetric assay, as described in our previous work [38]. Specifically, A431 cells were seeded in 96-well plates in the complete medium at a density of 6 × 10^3^ and kept in the humidified incubator for 24 h. Then, the cells were treated with 100 μΜ of the compounds (0.5% *v*/*v* DMSO) and incubated for 24 h. After incubation, 100 μL of the MTT solution (final concentration 0.65 mg/mL in the complete medium) was added in each well and the plate was incubated for 3 h. The medium was removed and 200 μL of DMSO was added in each well for the complete solubilization of the formazan crystals. The absorbance was measured at 570 nm using a BioTek Epoch 2 Microplate Spectrophotometer. The control samples contained only DMSO instead of the coumarin analogs. The results are expressed as % cytotoxicity = [1 − (mean optical density (OD) of treated cells/mean OD of untreated cells)] × 100. All measurements were carried out in triplicate.

## 4. Conclusions

In the present work, a ligand-free synthetic protocol for the synthesis of coumarin analogs via Suzuki–Miyaura coupling in NADES was developed, leading to the synthesis of ten novel coumarin derivatives (**4a**–**4j**). To our knowledge, a ligand-free synthetic procedure for the synthesis of coumarin derivatives via Suzuki–Miyaura coupling using NaDES is reported for the first time in the literature. Towards a more sustainable approach, five different NaDES as well as pure glycerol were examined as solvents, along with two inorganic bases (K_2_CO_3_, Na_2_CO_3_) and palladium catalysts (PdCl_2_, Pd(OAc)_2_). The highest yield (95%) was obtained when Choline chloride/Glycerol = 1:2 NaDES was used as a solvent along with K_2_CO_3_ and Pd(OAc)_2_. The change in the color of the reaction mixture indicated the formation of PdNPs during the reaction; a hypothesis which was assessed using Dynamic Light Scattering as well as Transmission Electron Microscopy. Interestingly, the size of the PdNPs can be associated with the reaction yield since the reaction proceeded smoothly in Choline chloride/Glycerol = 1:2 and Betaine/Glycerol = 1:2 NaDES in which nanoparticles of <140 nm were formed, whereas in L-proline/Glycerol = 1:2 NaDES and in pure glycerol, in which the size of the NPs was >467 nm, the reaction did not proceed at all. The TEM images revealed the formation of PdNPs of a spherical shape with a diameter of 5–10 nm in the ChCl/Gly = 1:2 NaDES. The recyclability and reusability of the ChCl/Gly = 1:2 NaDES was examined, and it was found that it can be effectively reused up to two times without a significant decrease in the reaction yield.

Interestingly, when a coumarin bearing an acetyloxy moiety was used, a concomitant removal of the acetyl group was succeeded under the reaction conditions. Thus, the carbon–carbon bond formation and the deacetylation of the coumarin analogs were achieved in one pot. The deacetylation of (4-acetyloxyphenyl)-6-bromo-4-methyl-coumarin **3b** was studied in both ChCl/Gly = 1:2 NaDES and in pure glycerol, using K_2_CO_3_ as the base, while applying conventional heating at 90 °C or ultrasound irradiation. The ultrasound irradiation led to significantly shorter reaction times. In the case when glycerol was used as the solvent, the deacetylation reaction under ultrasound irradiation was completed in only 6 min with a yield of 92%.

The novel multi-substituted coumarin analogs (**4a**–**4j**) that were synthesized were structurally characterized using 1D and 2D NMR spectroscopy and were examined for their cytotoxicity against the squamous carcinoma A431 cell line. It is noteworthy that 6 out of the 10 tested compounds exhibited cytotoxic activity higher than 50% at the concentration of 100 μΜ.

## Figures and Tables

**Table 1:** Tested conditions and yields of the model reaction.

Solvent *	Base	Catalyst (0.025 mol/mol Pd(II)/3b)	Yield (%)
Choline chloride/Glycerol = 1:2	Na_2_CO_3_	Pd(OAc)_2_	85
Betaine/Glycerol = 1:2	Na_2_CO_3_	Pd(OAc)_2_	70
Choline chloride/Glycerol = 1:2	K_2_CO_3_	Pd(OAc)_2_	95
Choline chloride/Glycerol = 1:2	K_2_CO_3_	PdCl_2_	83
Betaine/Glycerol = 1:2	K_2_CO_3_	Pd(OAc)_2_	71
L-proline/Glycerol = 1:2	K_2_CO_3_	Pd(OAc)_2_	traces
L-proline/Glycerol = 1:2	K_2_CO_3_	PdCl_2_	traces
Glycerol	K_2_CO_3_	Pd(OAc)_2_	traces
Fructose/urea/H_2_O = 1:1.5:1	K_2_CO_3_	Pd(OAc)_2_	No
Glucose/urea/H_2_O = 1:1.5:1	K_2_CO_3_	Pd(OAc)_2_	No

* The ratios refer to the molar ratio of the components.

**Table 2:** Average hydrodynamic diameter, PolyDispersity Index (PDI) and zeta potential of the PdNPs formed in different glycerol-based NaDES and glycerol (0.016 mmol Pd(OAc)_2_, 3.1 g solvent, t = 5 min, 90 °C); Yield of the Suzuki–Miyaura coupling product **4b**.

Solvent	Average Hydrodynamic Diameter (nm)	PDI	ζ-Potential (mV)	Yield of 4b (%)
Choline chloride/Glycerol = 1:2	140.6 ± 14.8	0.343 ± 0.06	−18.6 ± 2.5	82–95
Betaine/Glycerol = 1:2	128.4 ± 40.0	0.476 ± 0.07	−22.7 ± 2.5	70–71
L-proline/Glycerol = 1:2	467.1 ± 54.3	0.634 ± 0.05	−36.4 ± 4.9	traces
Glycerol	180 nm (25%) >1 μm (75%)	-	-	traces

**Table 3:** Tested conditions, reaction time and yield for the deacetylation reaction.

Reaction Conditions	Solvent	Heating Method	Time	Yield (%)
i	ChCl/Gly = 1:2	Conventional heating (90 °C)	3 h	79
ii	ChCl/Gly = 1:2	Ultrasound irradiation	15 min	73
iii	Glycerol	Conventional heating (90 °C)	3 h	72
iv	Glycerol	Ultrasound irradiation	6 min	92

**Table 4:** ^1^H and ^13^C NMR data of compounds **4i** and **4j** in DMSO-d_6_.

Position	4i		4j	
	δ_H_, Multi. (*J*)	δ_c_, Multi. (*J*)	δ_H_, Multi. (*J*)	δ_c_, Multi. (*J*)
1	-	-	-	-
2	-	160.1	-	160.2
3	-	126.8	-	126.4
4	-	147.5	-	147.6
CH_3_	2.38	16.7	2.39	16.5
4a	-	120.7	-	120.5
5	8.03, d (*J* = 1.8 Hz)	124.1	8.01, d (*J* = 1.8 Hz)	123.4
6	-	134.3	-	138.0
7	7.93, dd (*J* = 9.0 Hz, *J* = 1.8 Hz)	125.0	7.92, dd (*J* = 8.4 Hz, *J* = 2.1 Hz)	129.9
8	7.50, d (*J* = 9.0 Hz)	116.9	7.50, d (*J* = 8.7 Hz)	116.7
8a	-	151.8	-	150.9
1′	-	129.9	-	124.9
2′/6′	7.13, d (*J* = 7.2 Hz)	131.6	7.15, d (*J* = 8.4 Hz)	131.5
3′/5′	6.84, d (*J* = 7.2 Hz)	115.0	6.85, d (*J* = 8.4 Hz)	114.9
4′	9.65, s (OH)	157.3	9.63, s (OH)	157.1
1″	-	136.8	-	132.3
2″	7.95, dd (*J* = 7.8 Hz, *J* = 1.8 Hz)	116.2, d (^2^*J*_C-F_ = 17.8 Hz)	7.73, s	109.7
3″	-	150.4, dd (*J*_C-F_ = 89 Hz, ^2^*J*_C-F_ = 12.5 Hz)	-	136.5
4″	-	148.8, dd (*J*_C-F_ = 89 Hz, ^2^*J*_C-F_ = 12.5 Hz)	11.19, s (NH)	-
5″	7.55, dt (*J* = 8.4 Hz)	118.1, d (^2^*J*_C-F_ = 17 Hz)	7.41–7.39, m	126.4
6″	7.65 br	123.8, dd (^3^*J*_C-F_ = 6.5 Hz, ^4^*J*_C-F_ = 3 Hz)	6.47, br	101.0
7″	-	-	-	127.3
8″	-	-	7.65, d (*J* = 8.1 Hz)	120.5
9″	-	-	7.41–7.39, m	118.5

**Table 5:** Cytotoxicity results of the novel coumarin analogs against the A431 squamous cancer cell line.

Compound	Structure	Cytotoxicity (%) (100 μM)
**3b**	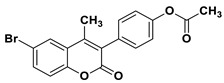	22.1 ± 5.5
**5**	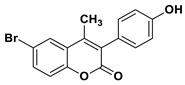	58.1 ± 1.0
**4a**	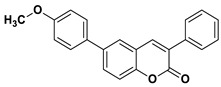	59.3 ± 1.4
**4b**	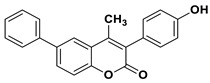	57.9 ± 0.7
**4c**	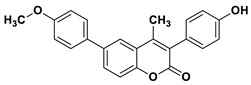	17.5 ± 1.7
**4d**	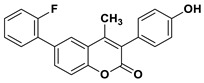	No
**4e**	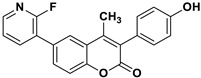	36.4 ± 1.5
**4f**	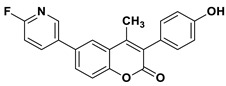	n.t.
**4g**	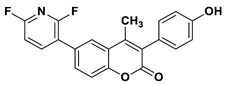	69.9 ± 4.3
**4h**	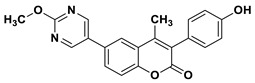	No
**4i**	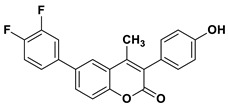	67.9 ± 0.3
**4j**	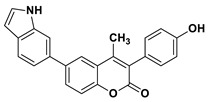	52.7 ± 2.5

No: No activity. n.t.: not tested.

## Data Availability

Data are contained within the article and Supplementary Materials.

## Supplementary Materials

The following supporting information can be downloaded at: https://www.mdpi.com/article/10.3390/molecules29184398/s1. Figures S1–S30: Nuclear Magnetic Resonance data; Figures S31–S40: HR-MS data.
