# Supplementary material for: A Ligand-Free Approach towards Coumarin Analogs via Natural Deep Eutectic Solvent-Mediated Suzuki–Miyaura Coupling
Source: Molecules. 2024 Sep 16;29(18):4398. doi: 10.3390/molecules29184398 (PMC11434556; doi:10.3390/molecules29184398)
Supplement: Supplementary file 1 [file molecules-29-04398-s001.zip › molecules-3144488-supplementary.pdf]

Supporting Information

A Ligand-free approach towards coumarin analogs via Natural Deep Eutectic Solvent-mediated Suzuki-Miyaura coupling

Annita Katopodi[1], Nikolaos Nikolaou[1], Eleni Alexandratou[2], Manolis Matzapetakis[3], Maria Zervou[3], Anastasia Detsi[1*]

[1]Laboratory of Organic Chemistry, School of Chemical Engineering, National Technical University of Athens, Zografou Campus, 15780, Athens, Greece
[2]Laboratory of Biomedical Optics and Applied Biophysics, School of Electrical and Computer Engineering, National Technical University of Athens, Zografou Campus, 15780 Athens, Greece
[3]Institute of Chemical Biology, National Hellenic Research Foundation, 48 Vas. Constantinou Ave., 11635, Athens, Greece

*Author for correspondence: adetsi@chemeng.ntua.gr

Nuclear Magnetic Resonance data

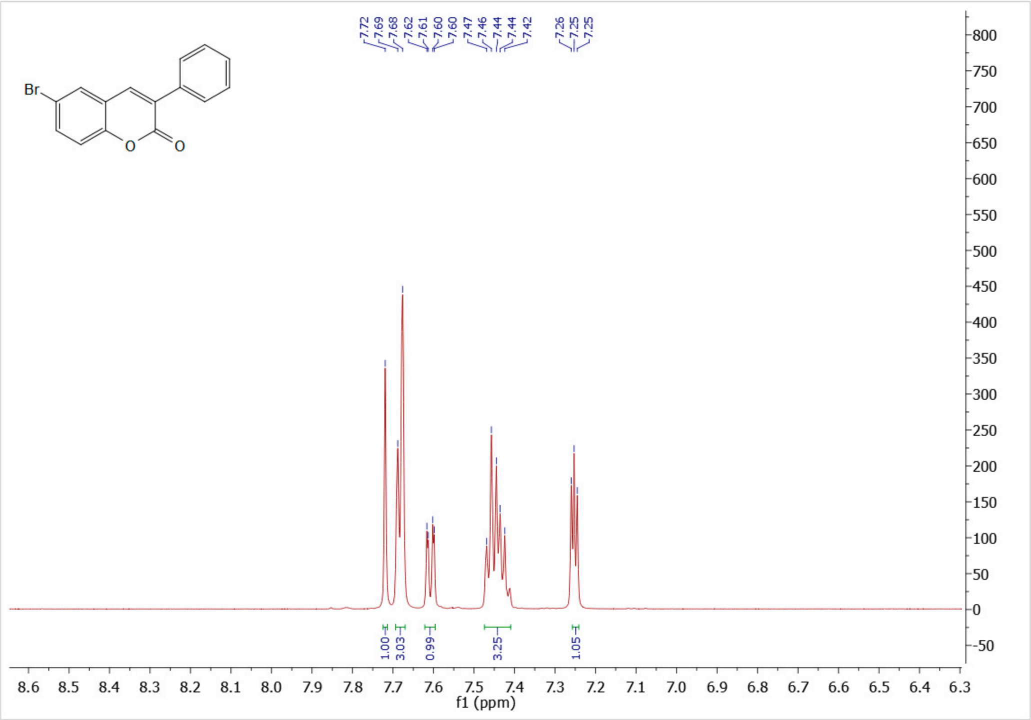

Figure S1. ^{1}H NMR spectrum of **3a** (600 MHz, $CDCl_3$).

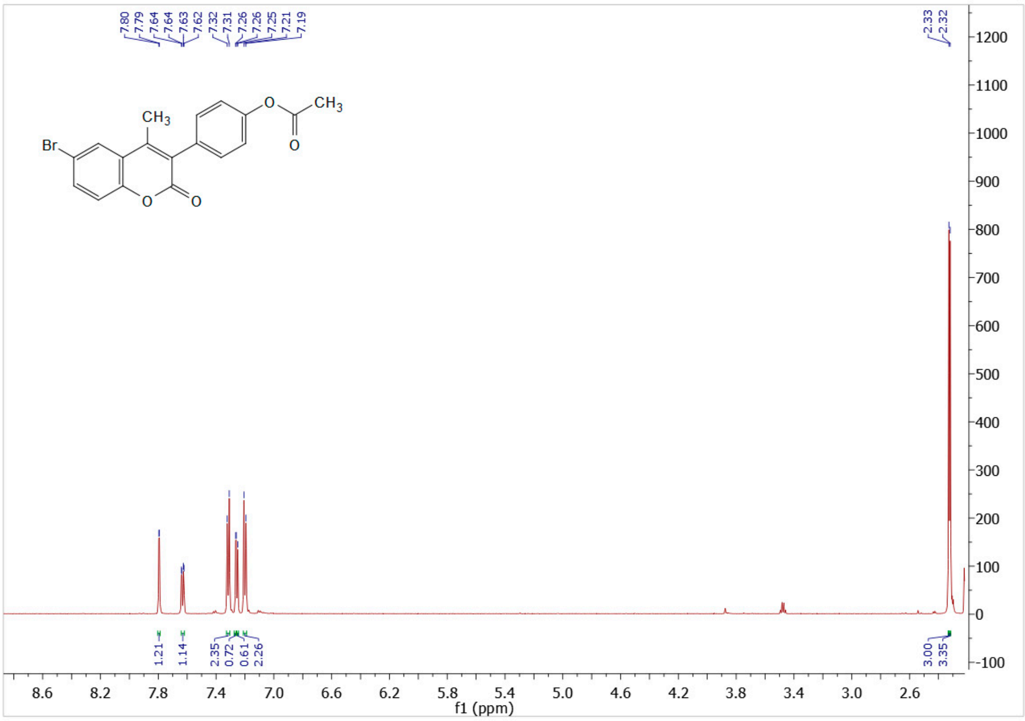

Figure S2. ^{1}H NMR spectrum of **3b** (600 MHz, $CDCl_3$).

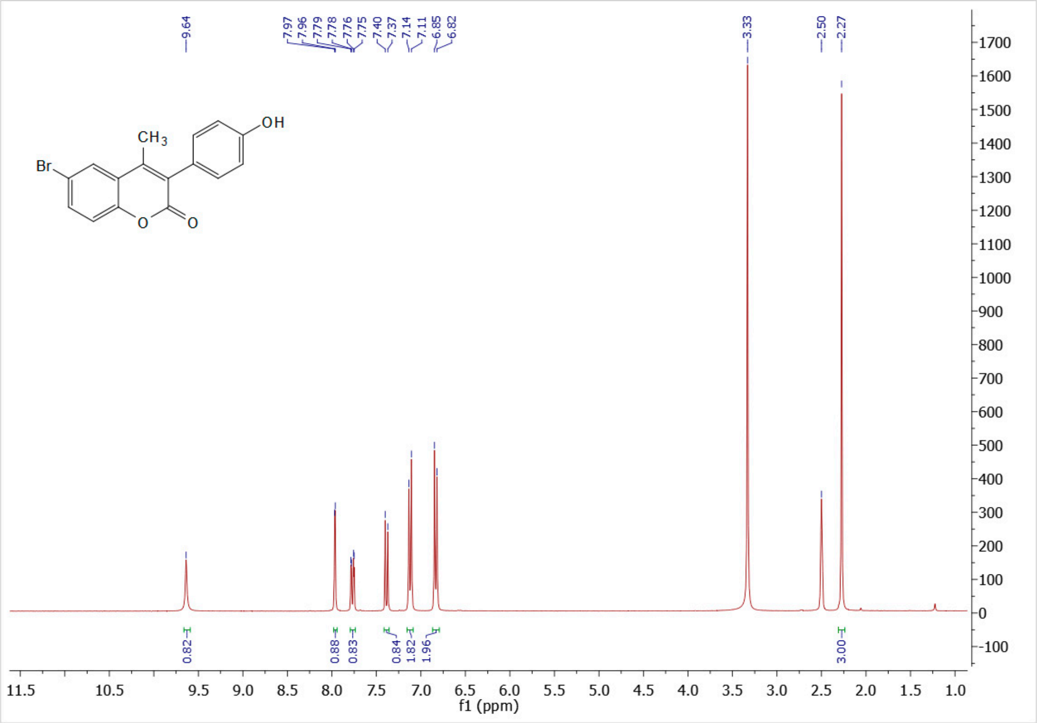

Figure S3. ^{1}H NMR spectrum of **5** (300 MHz, $CDCl_3$).

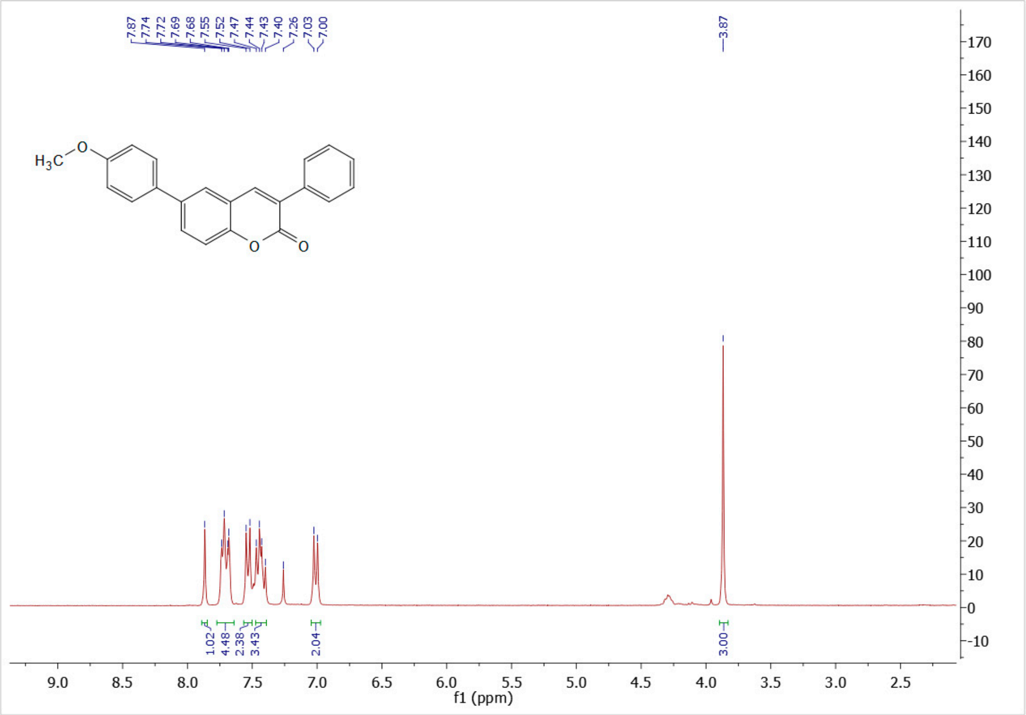

Figure S4. ^{1}H NMR spectrum of **4a** (300 MHz, DMSO-d_6).

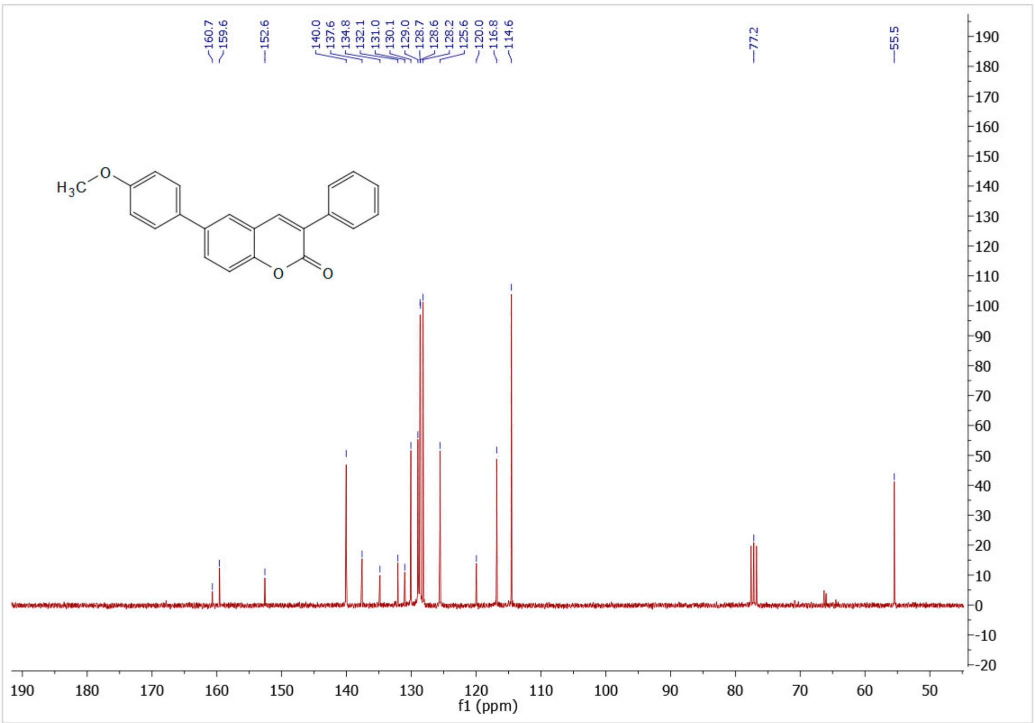

Figure S5. ^{13}C NMR spectrum of **4a** (75 MHz, $CDCl_3$).

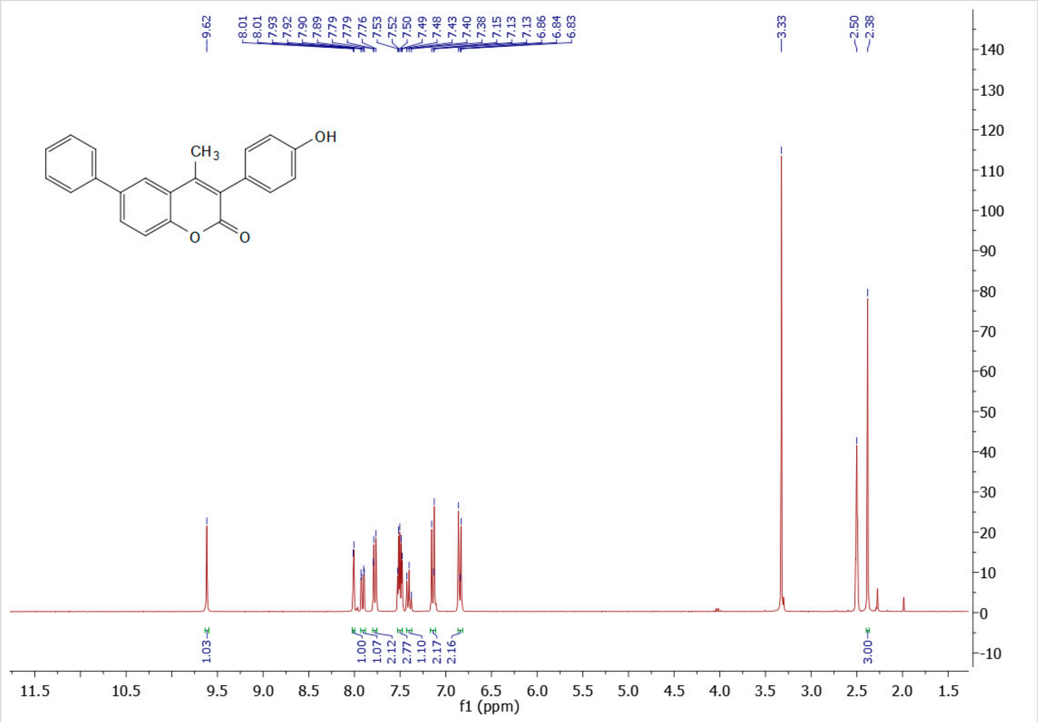

Figure S6. ^{1}H NMR spectrum of **4b** (600 MHz, DMSO-d_6).

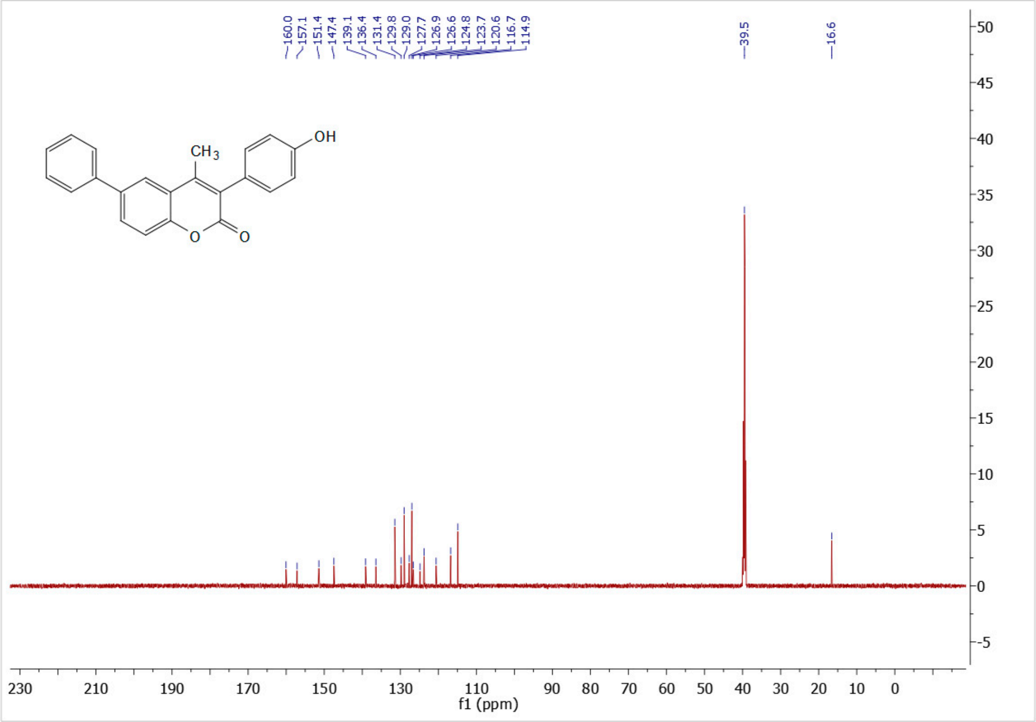

Figure S7. ^{13}C NMR spectrum of **4b** (150 MHz, DMSO-d_6).

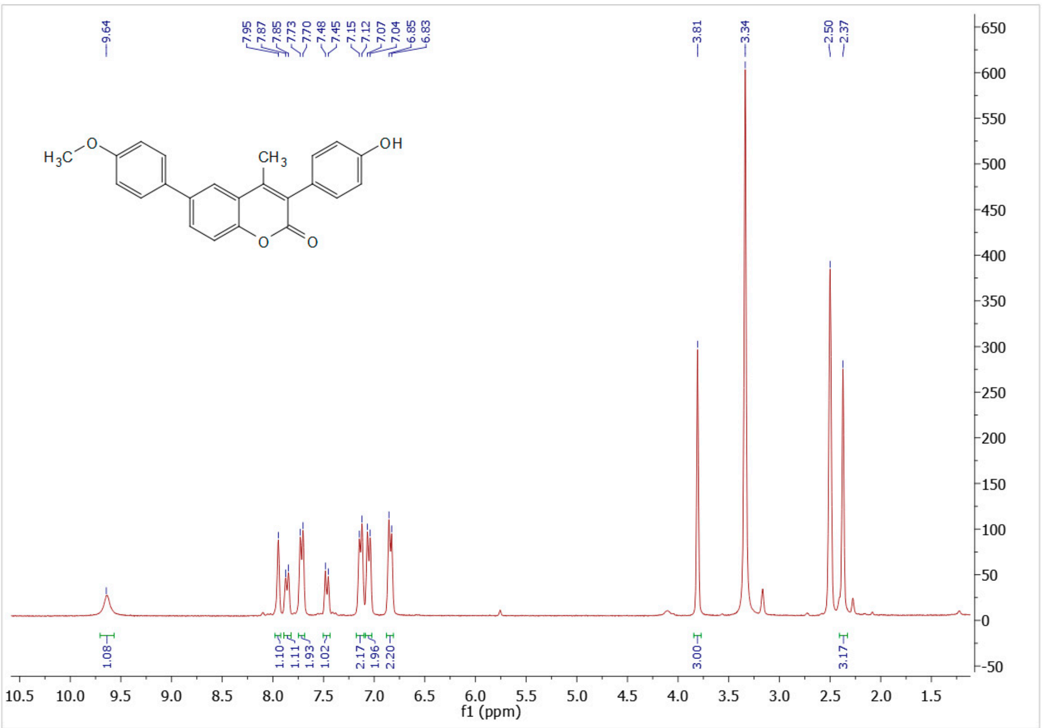

Figure S8. ^{1}H NMR spectrum of **4c** (600 MHz, DMSO-d_6).

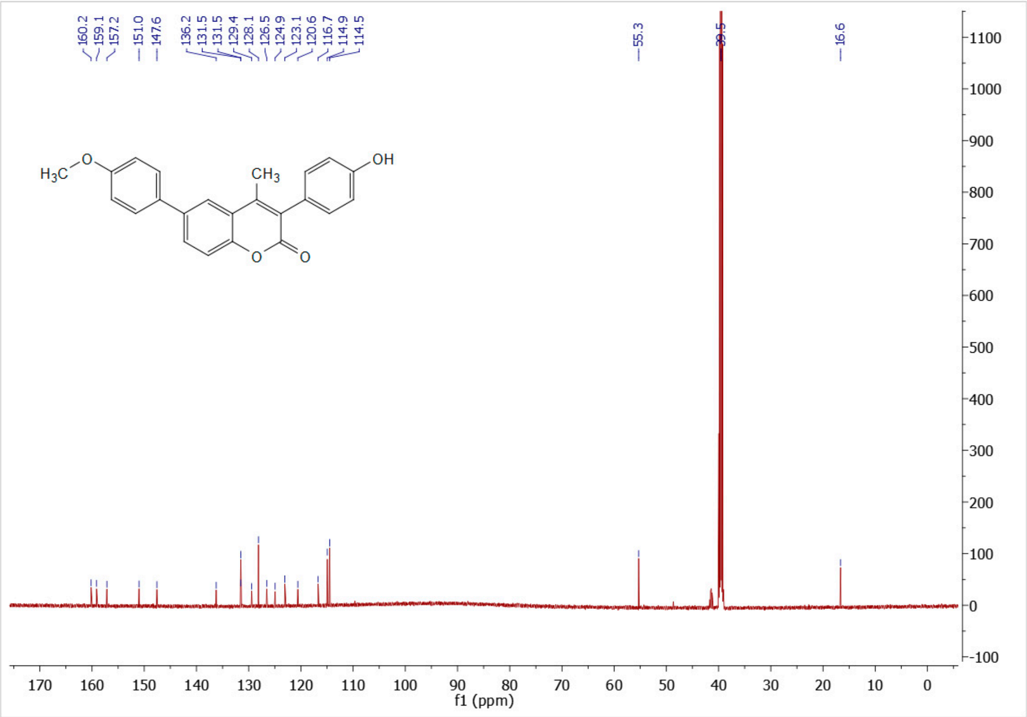

Figure S9. ^{13}C NMR spectrum of **4c** (150 MHz, DMSO-d_6).

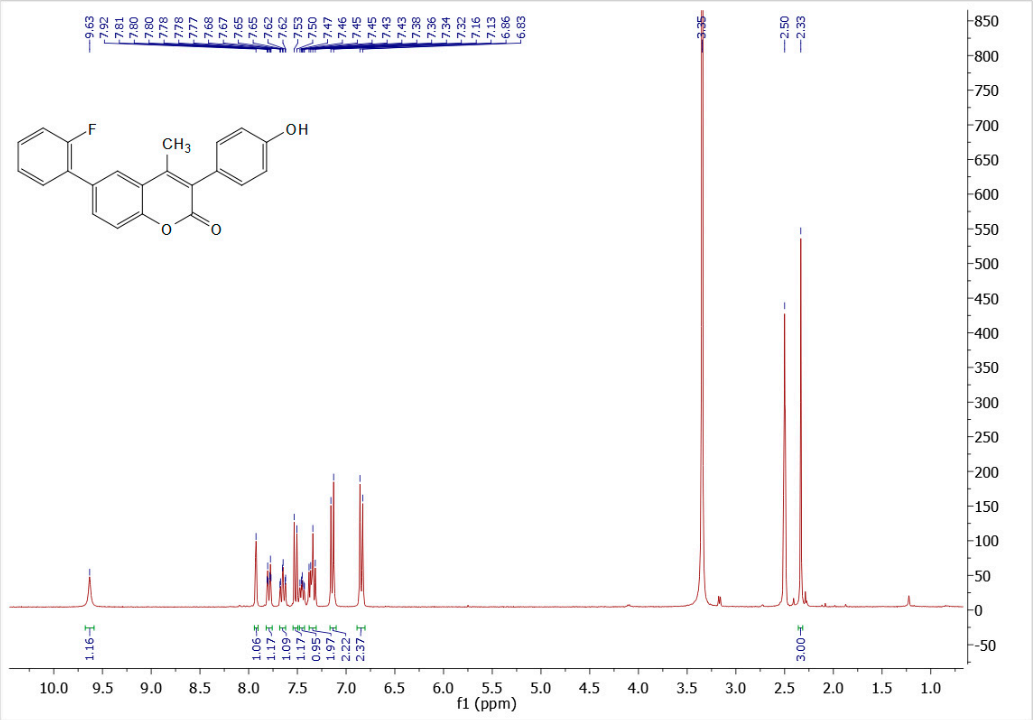

Figure S10. ^{1}H NMR spectrum of **4d** (300 MHz, DMSO-d_6).

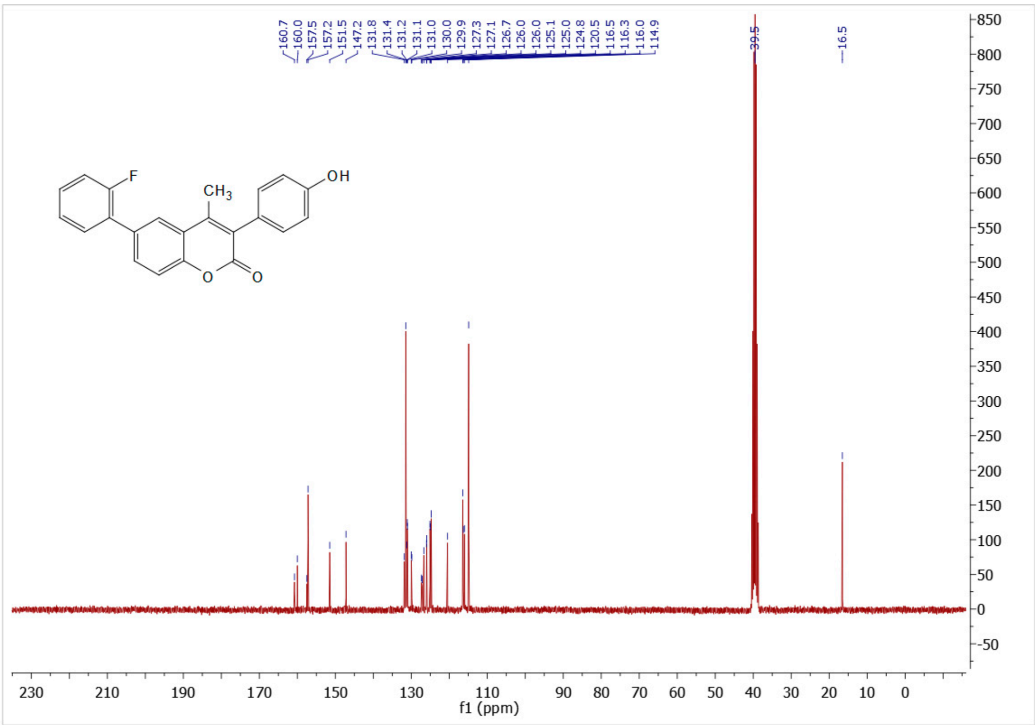

Figure S11. ^{13}C NMR spectrum of **4d** (75 MHz, DMSO-d$_6$).

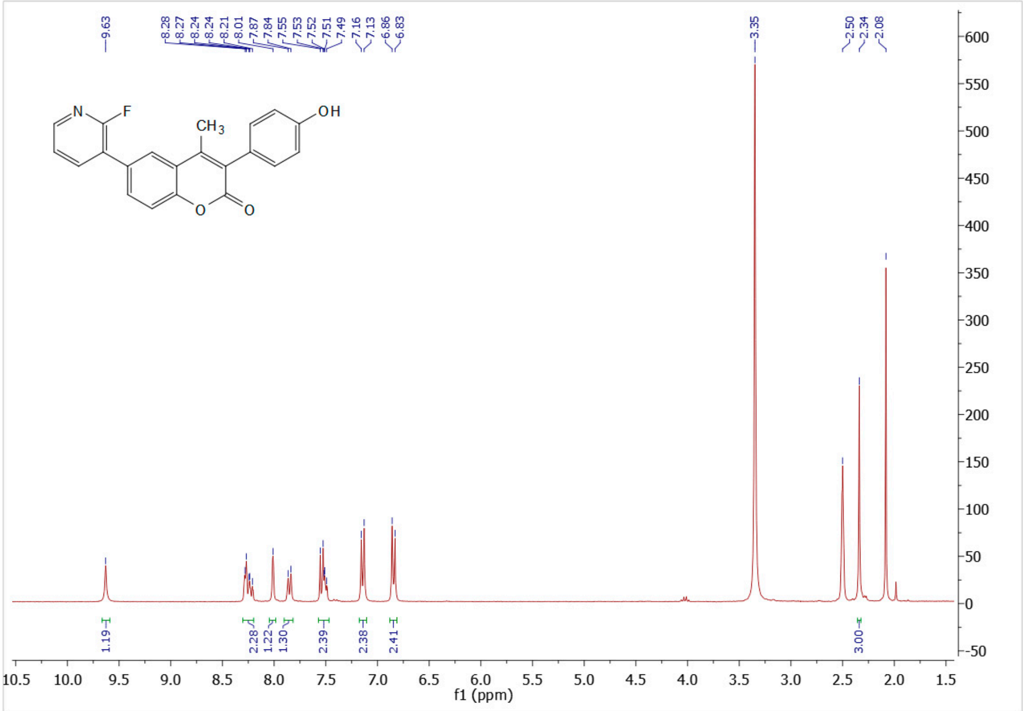

Figure S12. ^{1}H NMR spectrum of **4e** (300 MHz, DMSO-d$_6$).

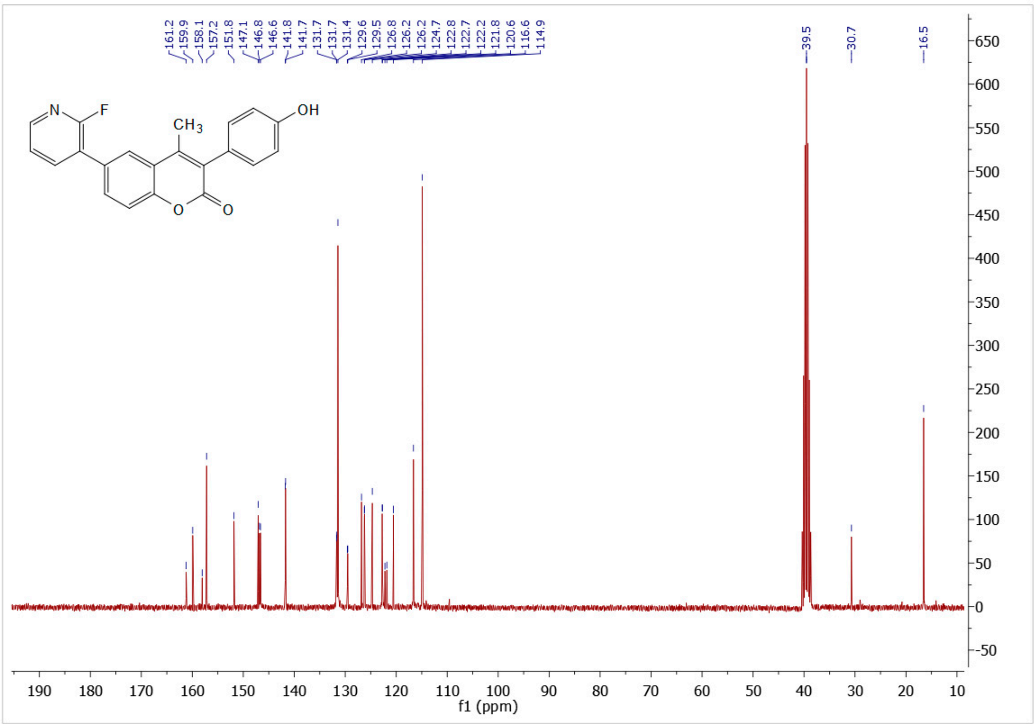

Figure S13. ^{13}C NMR spectrum of **4e** (75 MHz, DMSO-d_6).

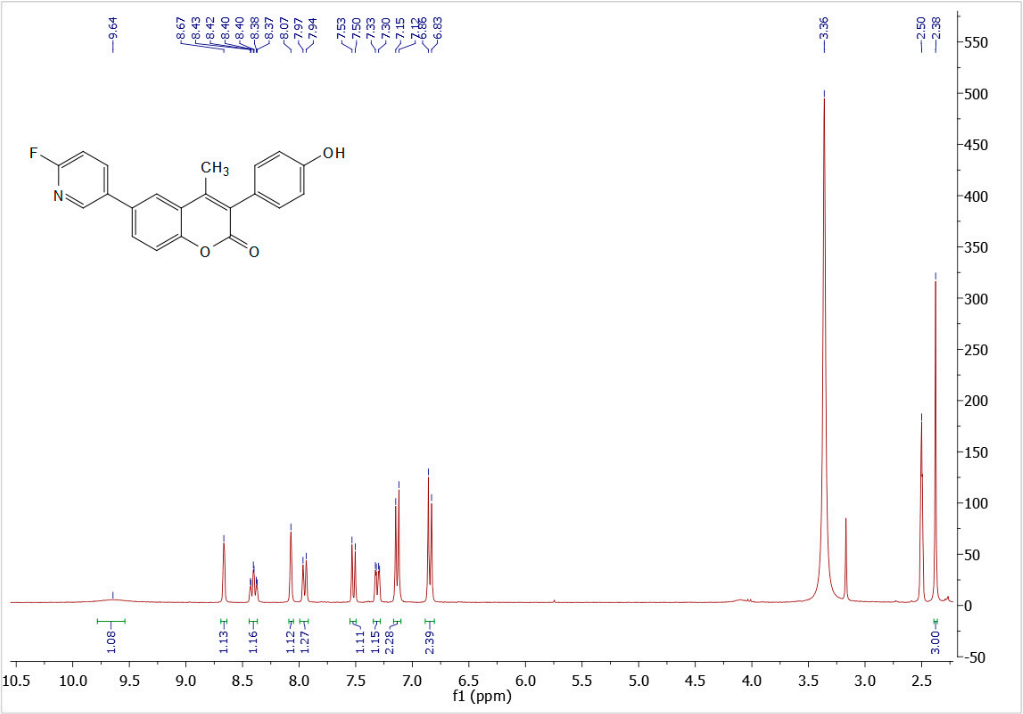

Figure S14. ^{1}H NMR spectrum of **4f** (300 MHz, DMSO-d_6).

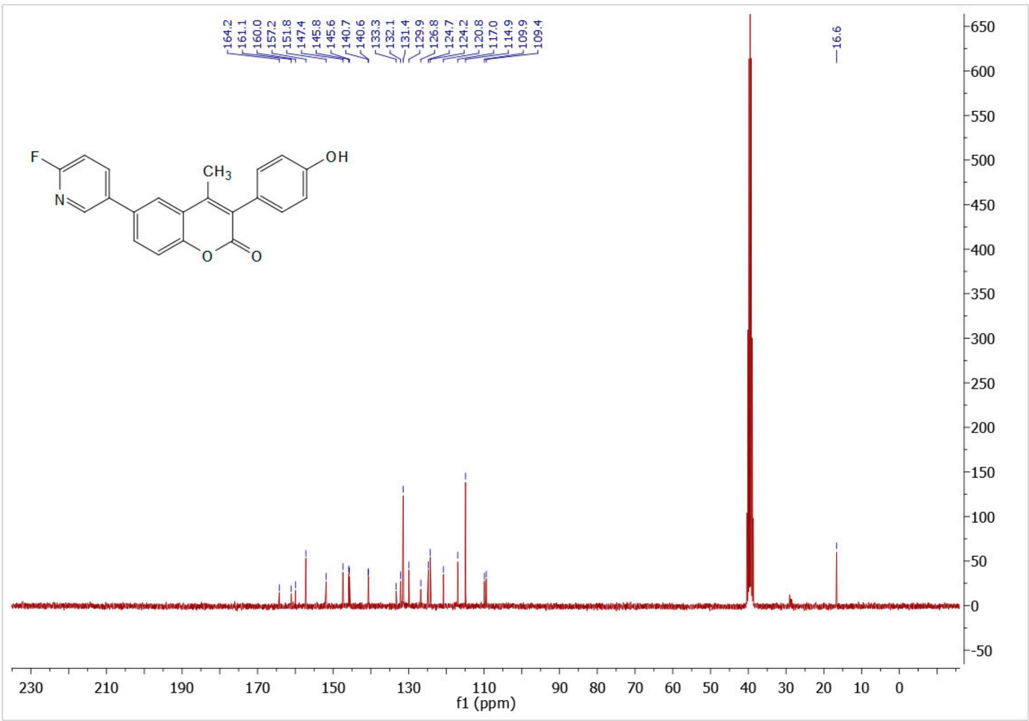

Figure S15. ^{13}C NMR spectrum of **4f** (75 MHz, DMSO-d$_6$).

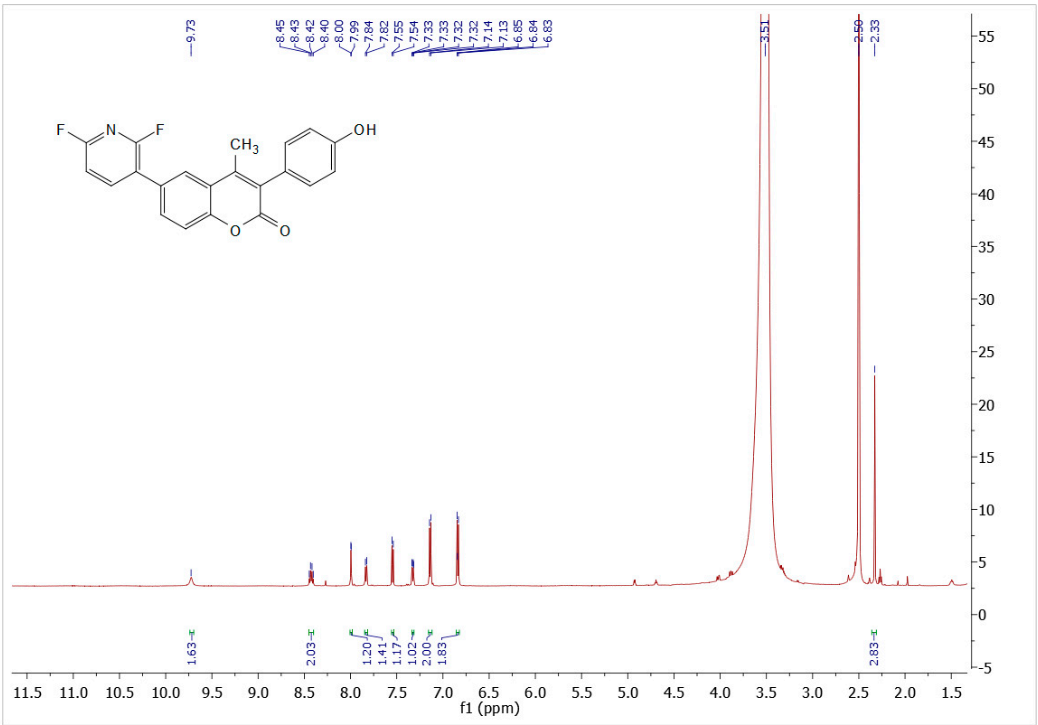

Figure S16. ^{1}H NMR spectrum of **4g** (600 MHz, DMSO-d$_6$).

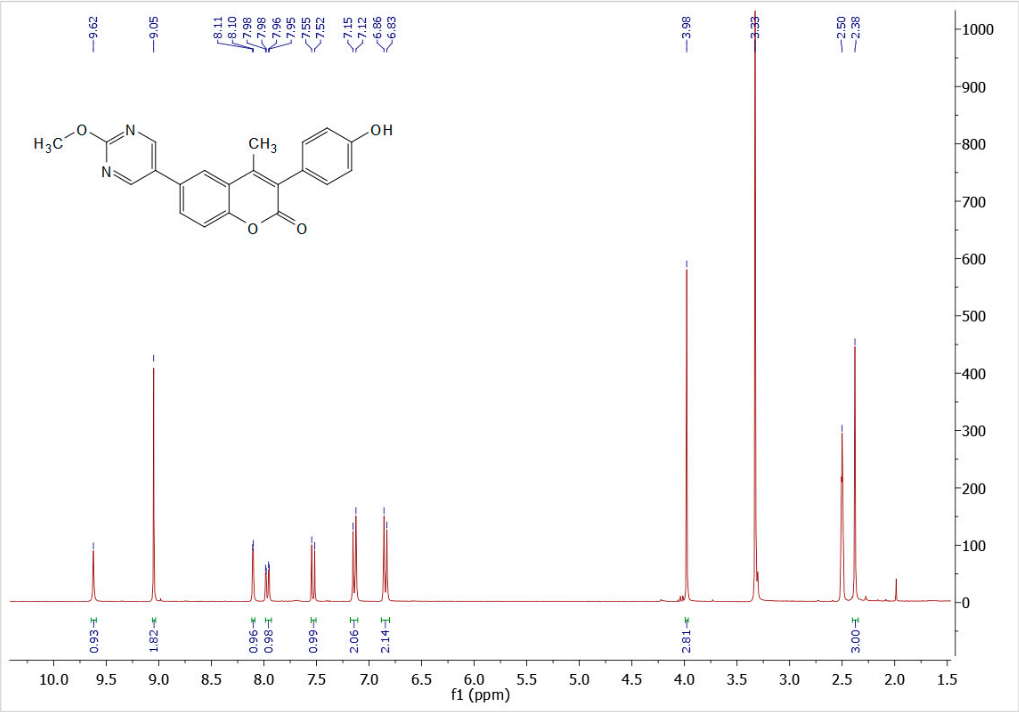

Figure S17. ^{1}H NMR spectrum of **4h** (300 MHz, DMSO-d_6).

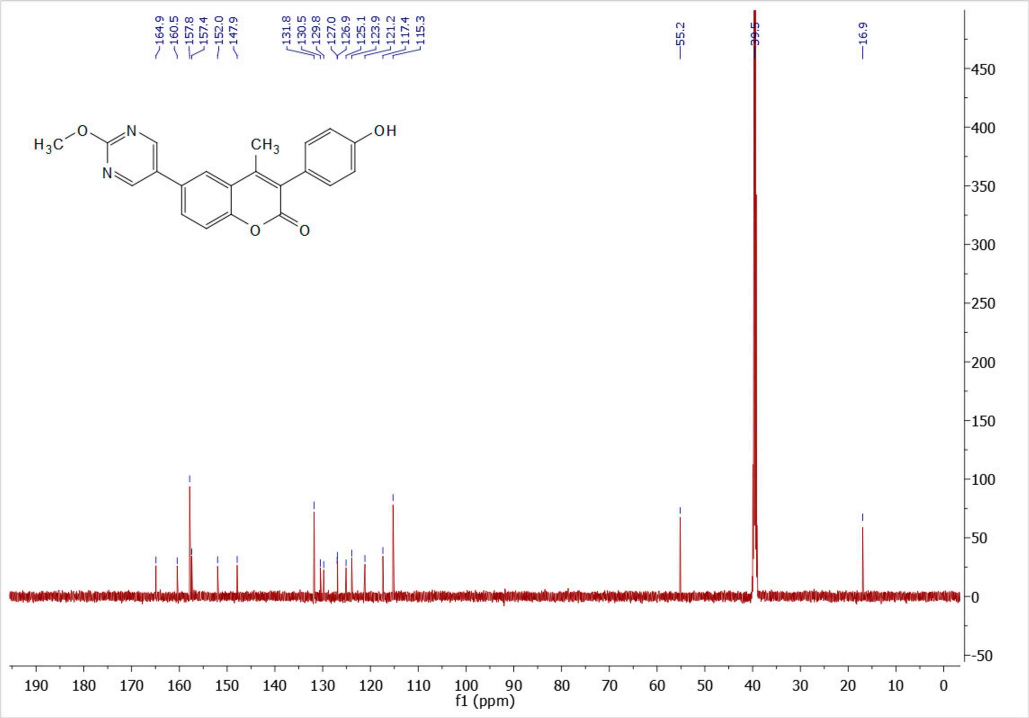

Figure S18. ^{13}C NMR spectrum of **4h** (150 MHz, DMSO-d_6).

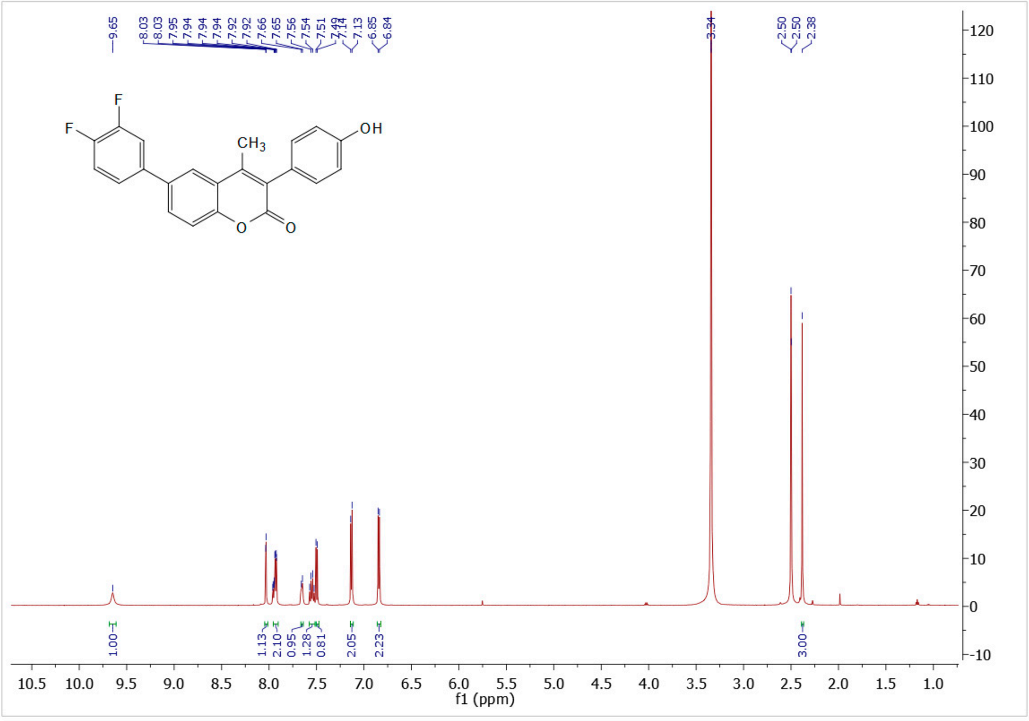

Figure S19. ^{1}H NMR spectrum of **4i** (600 MHz, DMSO-d_6).

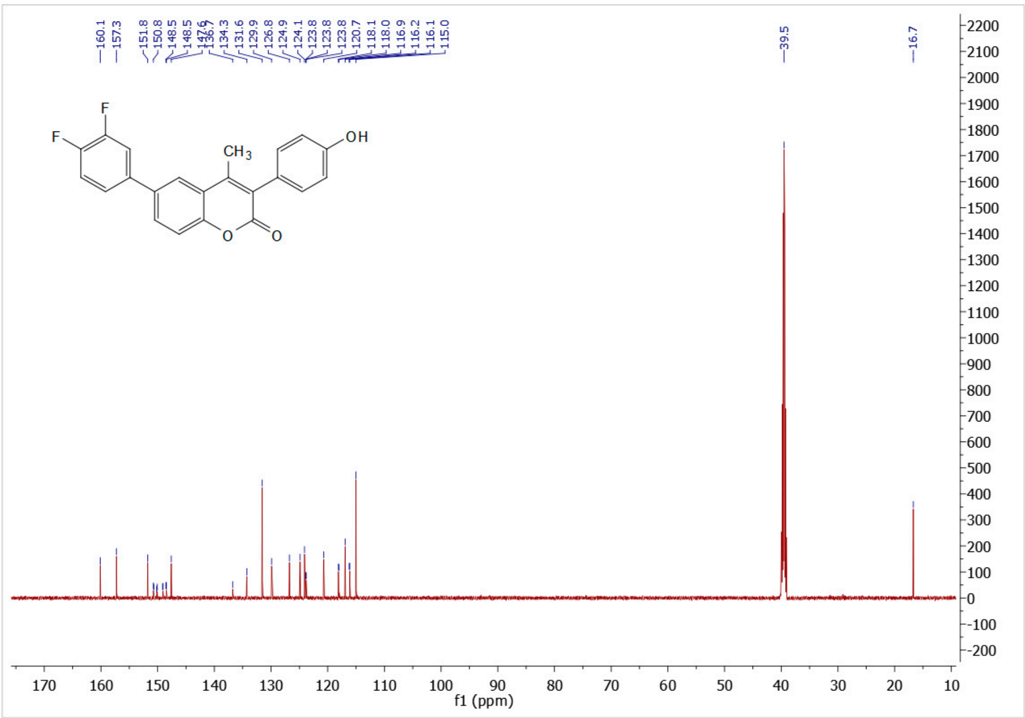

Figure S20. ^{13}C NMR spectrum of **4i** (150 MHz, DMSO-d_6).

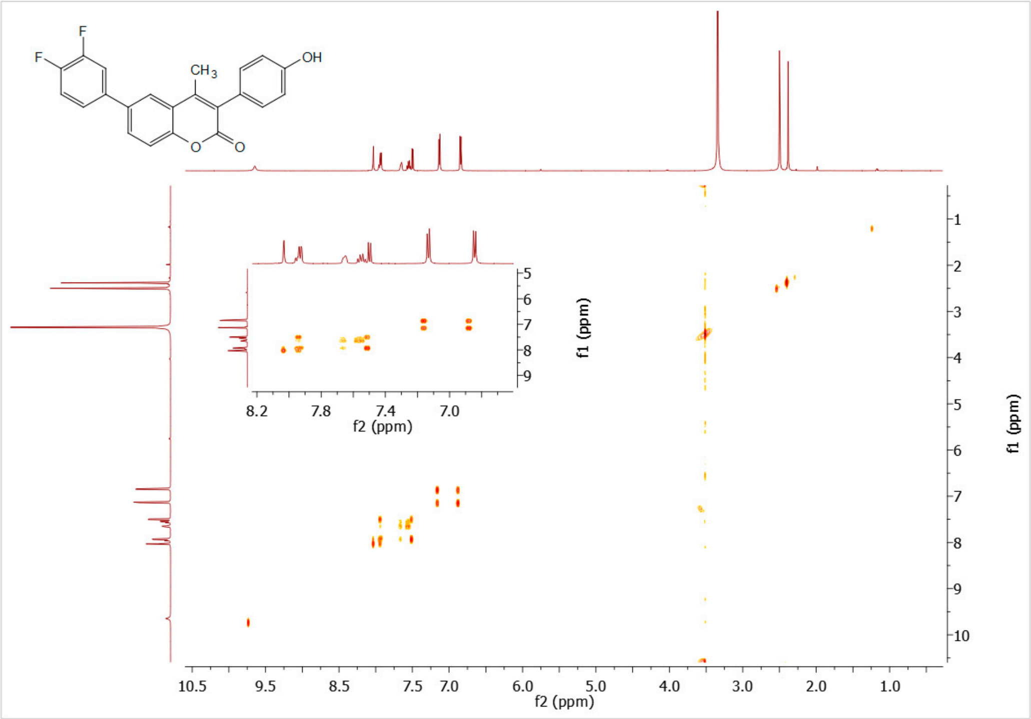

Figure S21. ^{1}H-^{1}H COSY NMR spectrum of **4i** (DMSO-d_6).

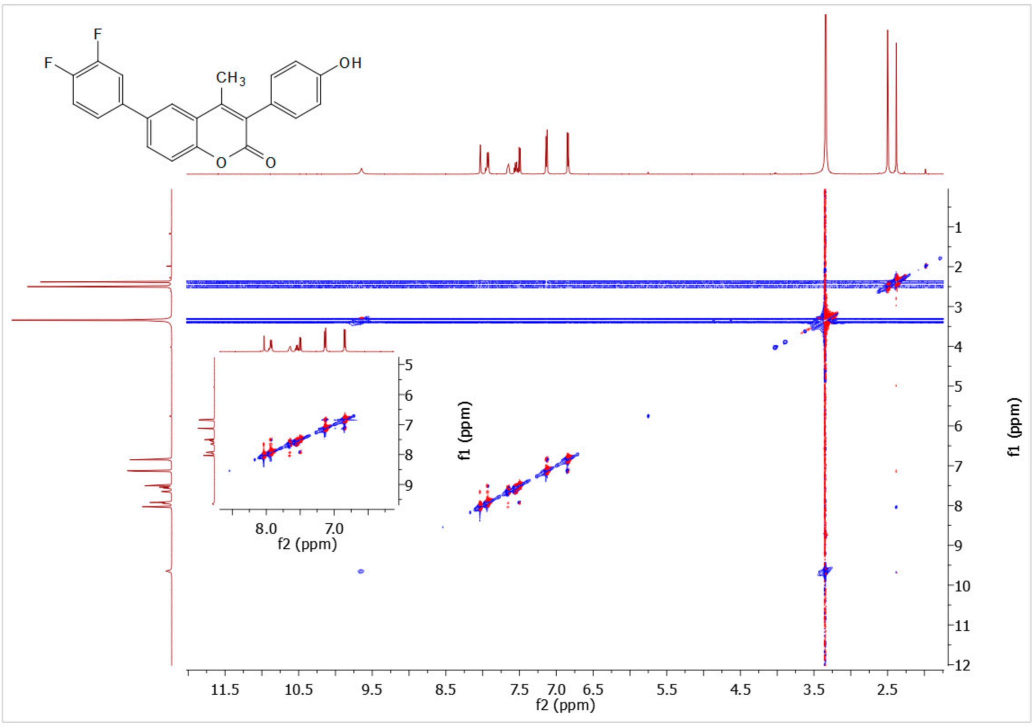

Figure S22. ^{1}H-^{1}H NOESY NMR spectrum of **4i** (DMSO-d_6).

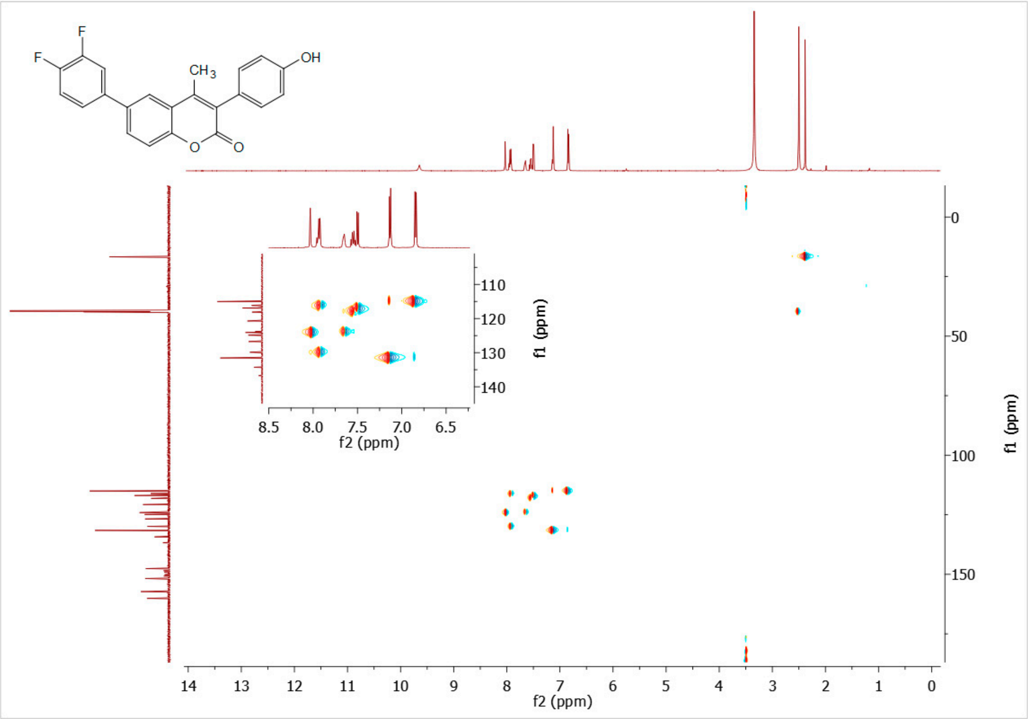

Figure S23. ^{1}H-^{13}C HSQC NMR spectrum of **4i** (DMSO-d_6).

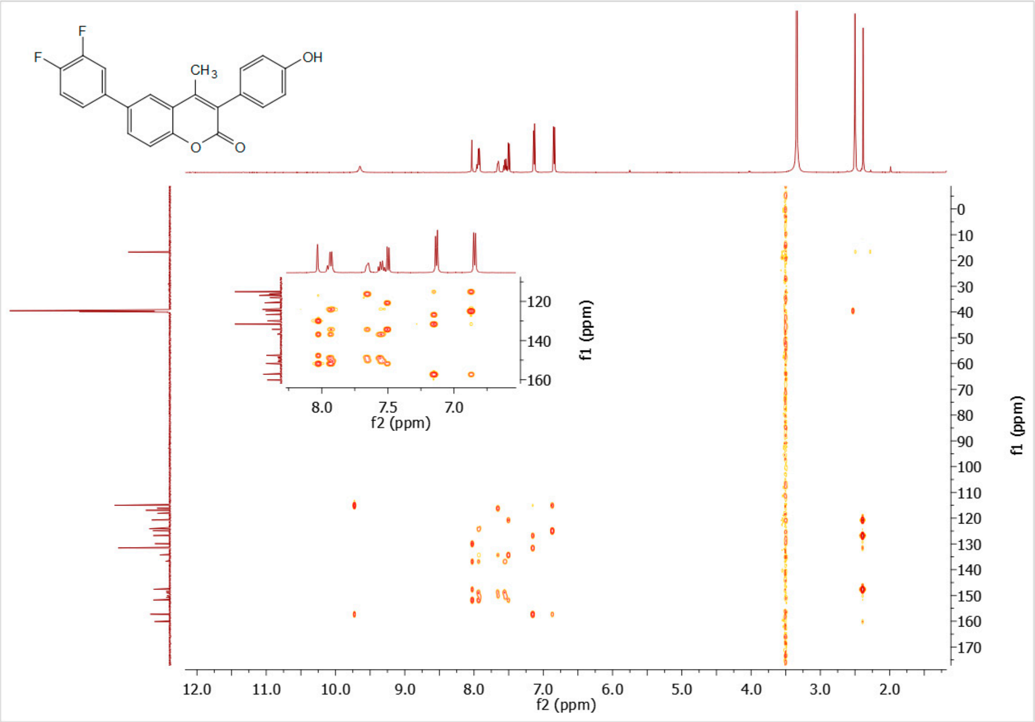

Figure S24. ^{1}H-^{13}C HMBC NMR spectrum of **4i** (DMSO-d_6).

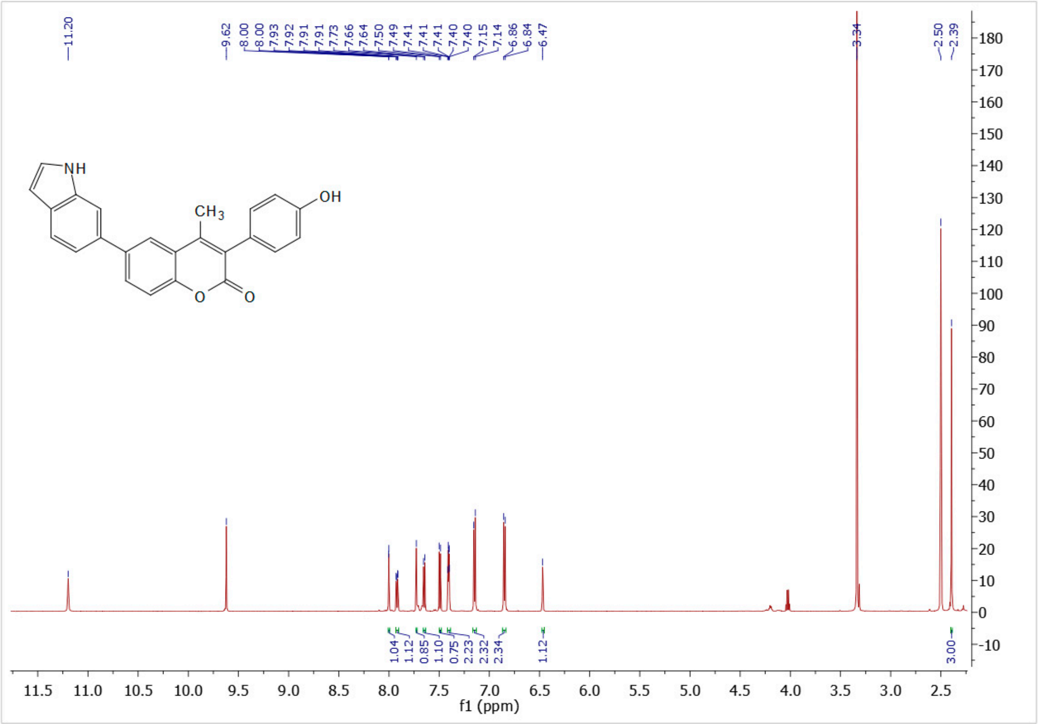

Figure S25. ^{1}H NMR spectrum of **4j** (600 MHz, DMSO-d_6).

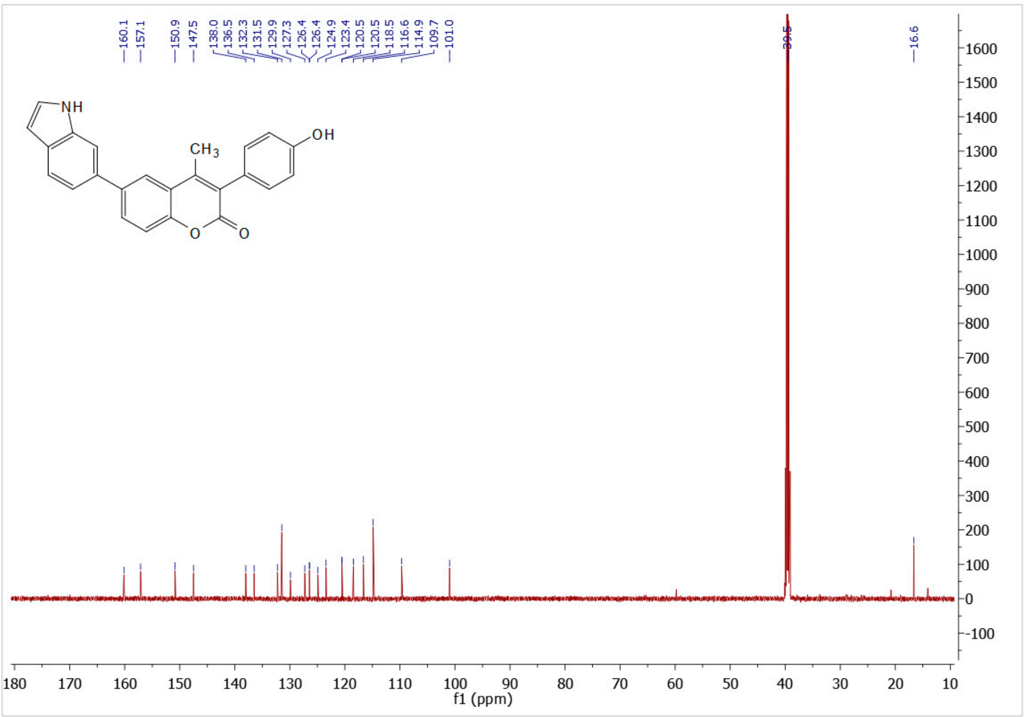

Figure S26. ^{13}C NMR spectrum of **4j** (150 MHz, DMSO-d_6).

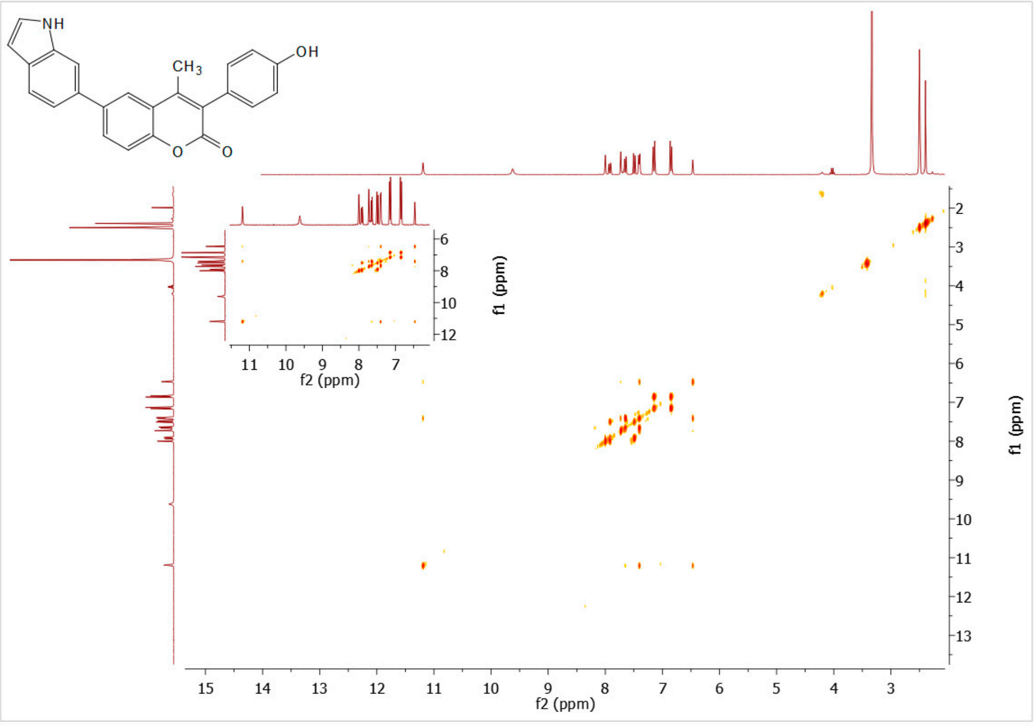

Figure S27. ^{1}H-^{1}H COSY NMR spectrum of **4j** (DMSO-d_6).

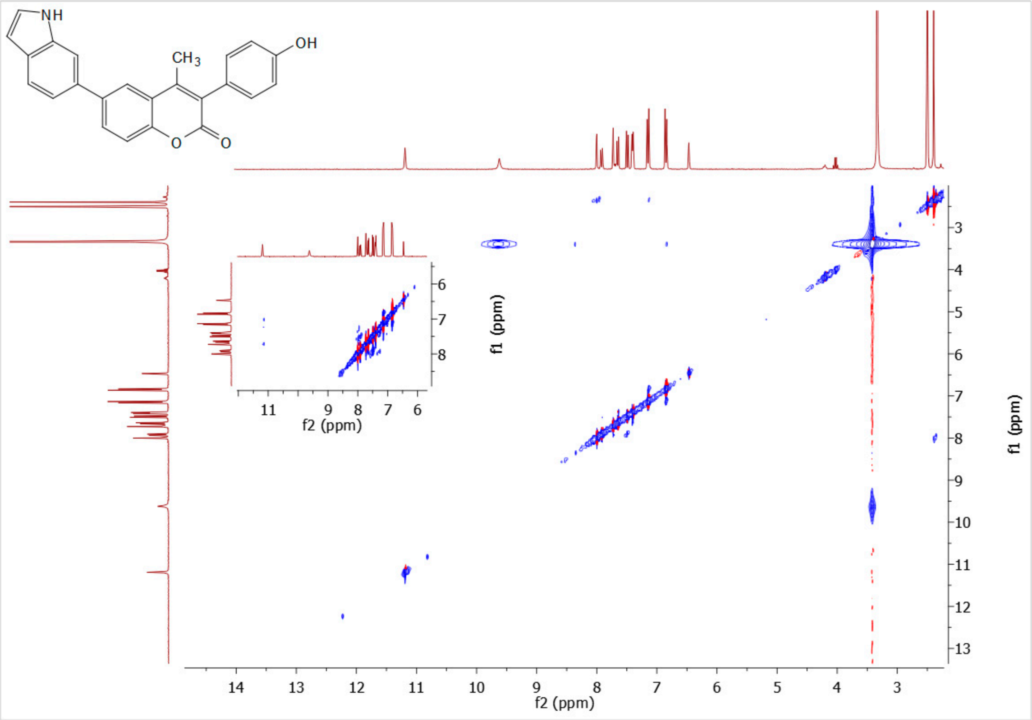

Figure S28. ^{1}H-^{1}H NOESY NMR spectrum of **4j** (DMSO-d_6).

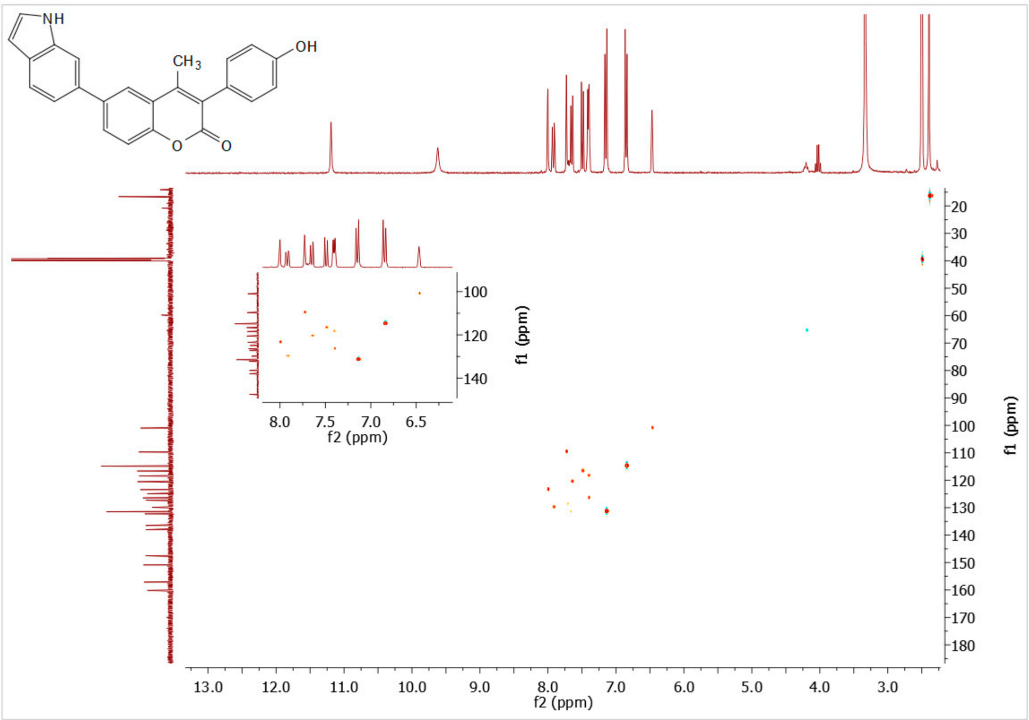

Figure S29. ^{1}H-^{13}C HSQC NMR spectrum of **4j** (DMSO-d_6).

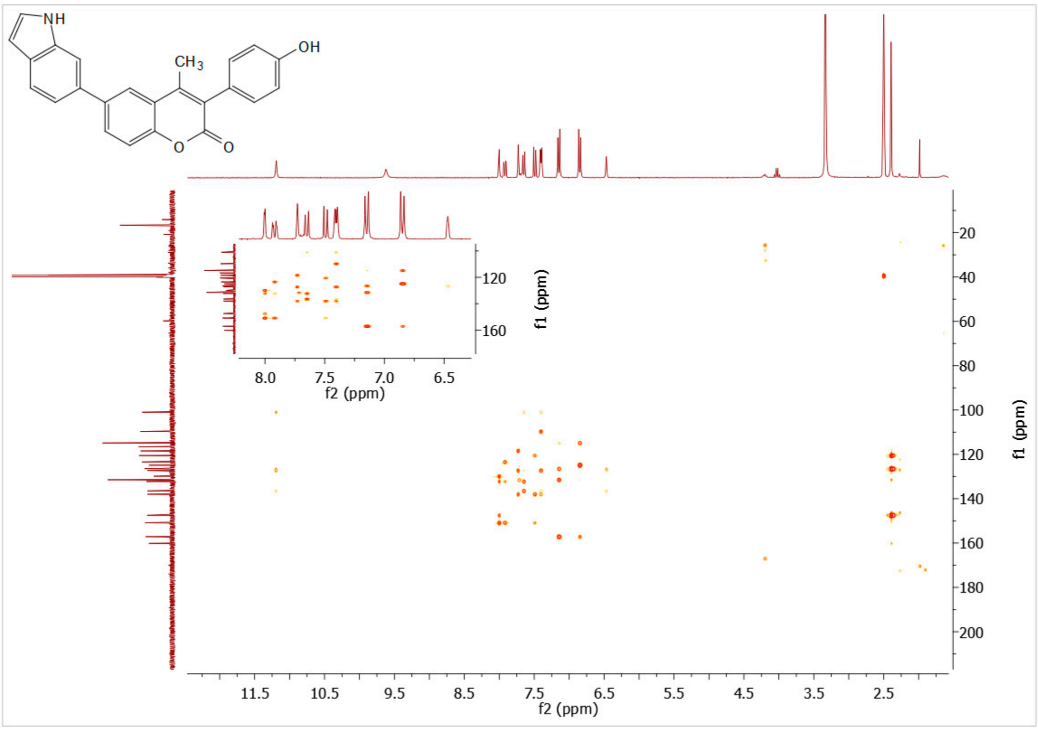

Figure S30. ^{1}H-^{13}C HMBC NMR spectrum of **4j** (DMSO-d_6).

HR-MS data

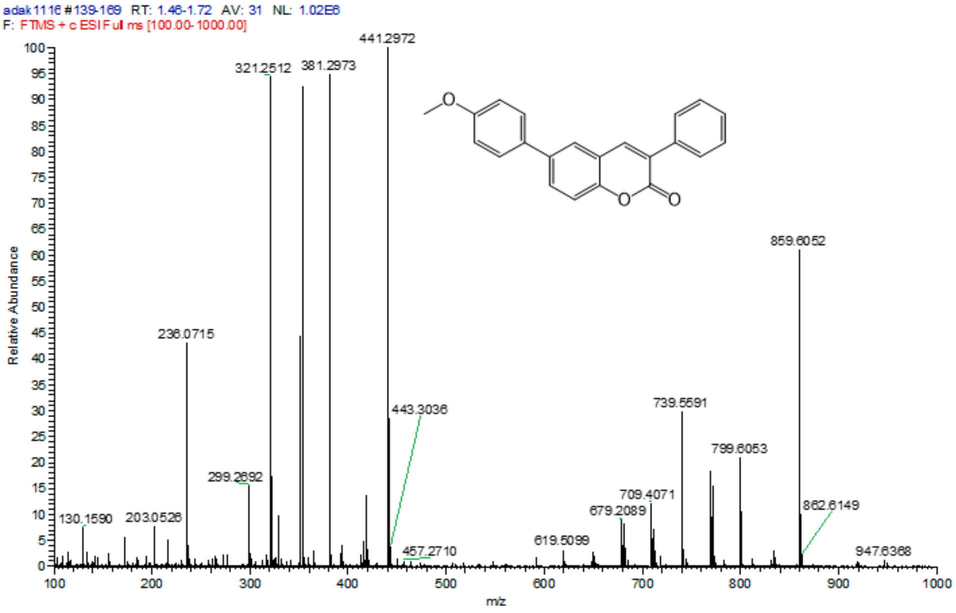

Figure S31. HR-MS spectrum of **4a**.

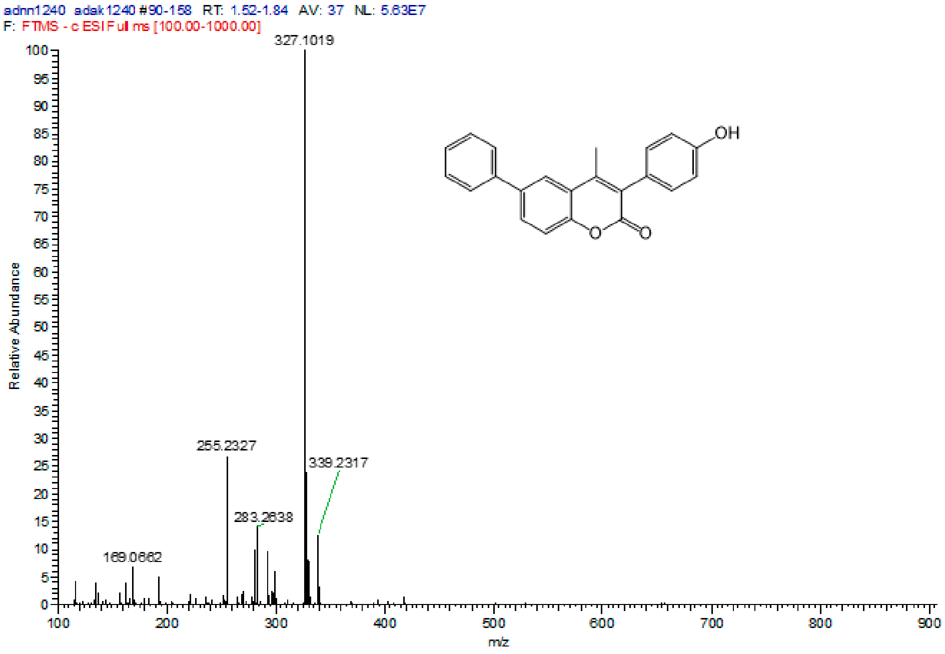

Figure S32. HR-MS spectrum of **4b**.

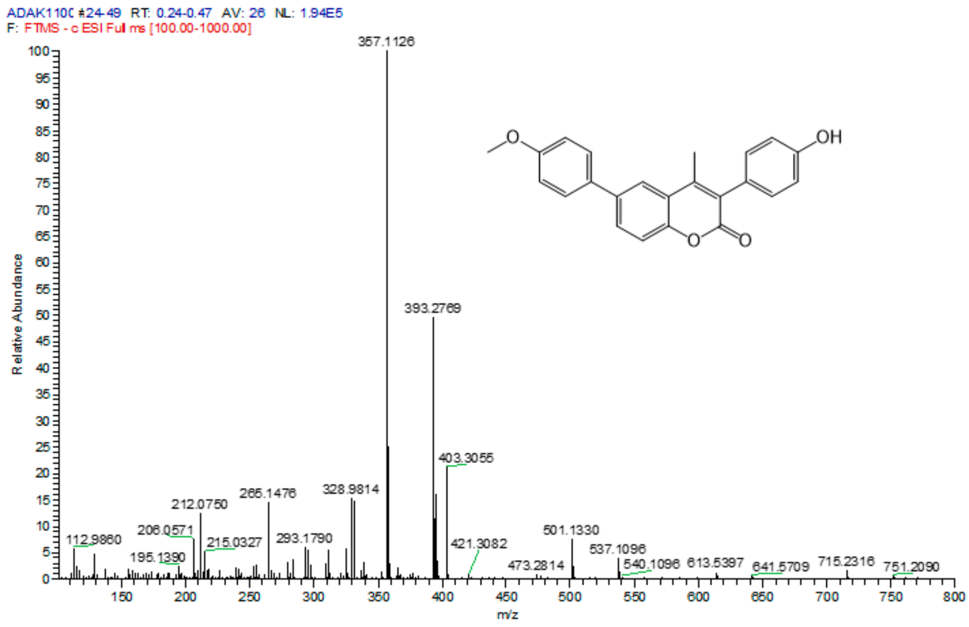

Figure S33. HR-MS spectrum of **4c**.

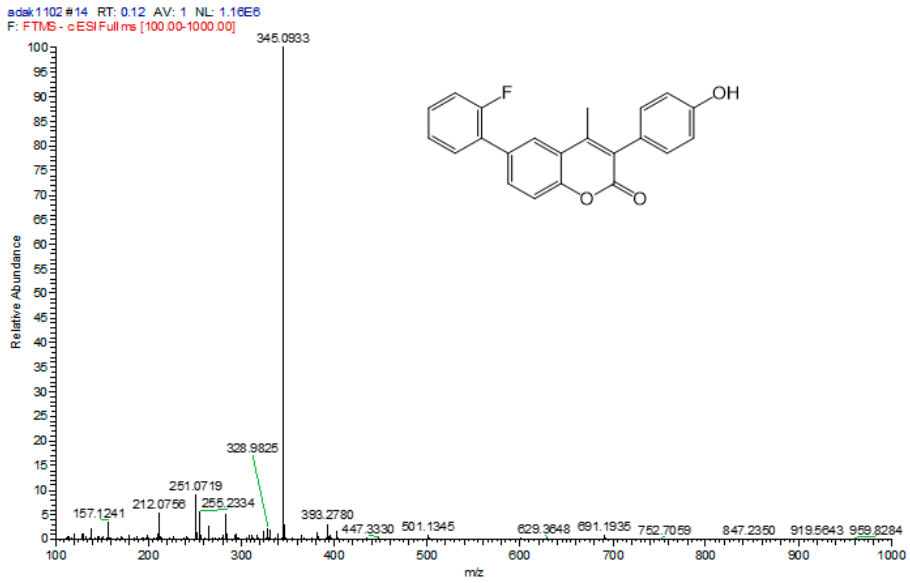

Figure S34. HR-MS spectrum of **4d**.

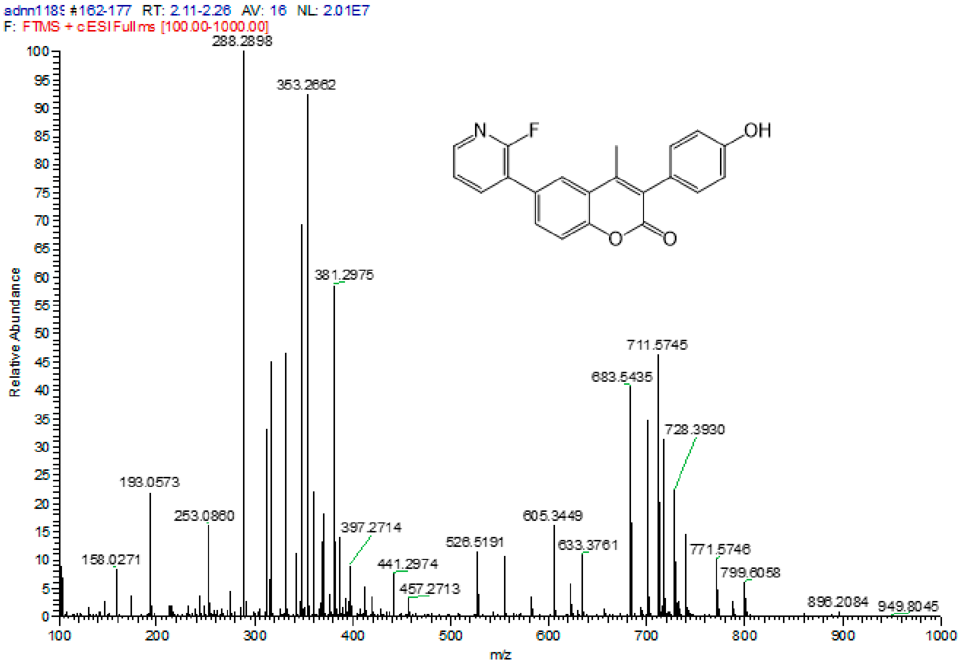

Figure S35. HR-MS spectrum of **4e**.

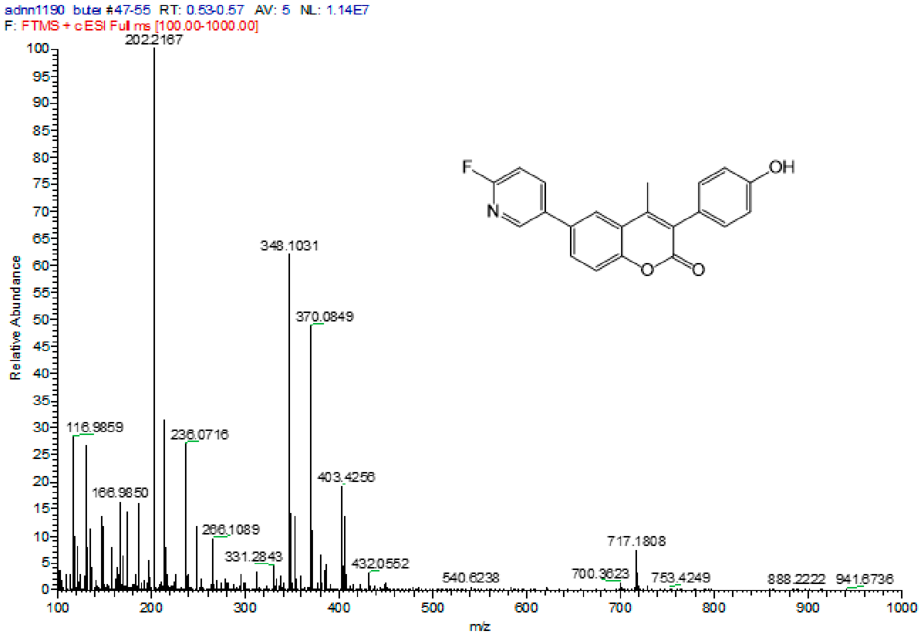

Figure S36. HR-MS spectrum of **4f**.

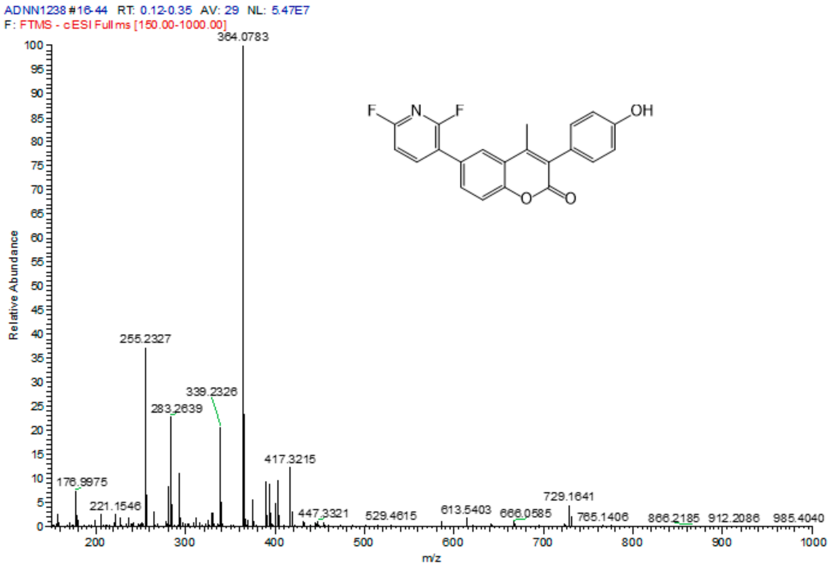

Figure S37. HR-MS spectrum of **4g**.

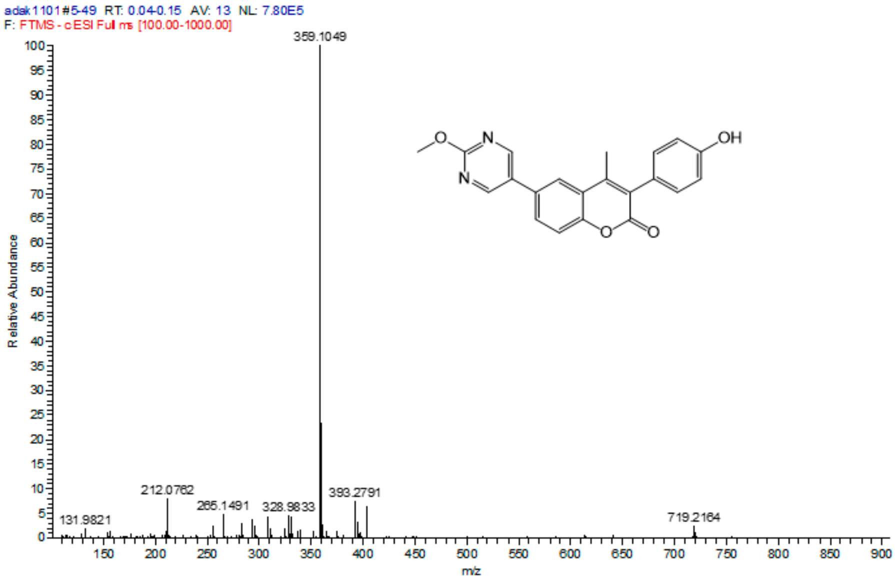

Figure S38. HR-MS spectrum of **4h**.

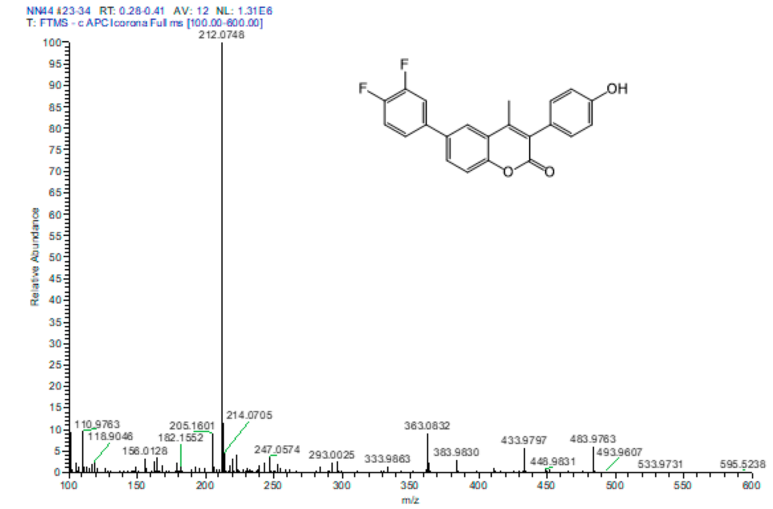

Figure S39. HR-MS spectrum of **4i**.

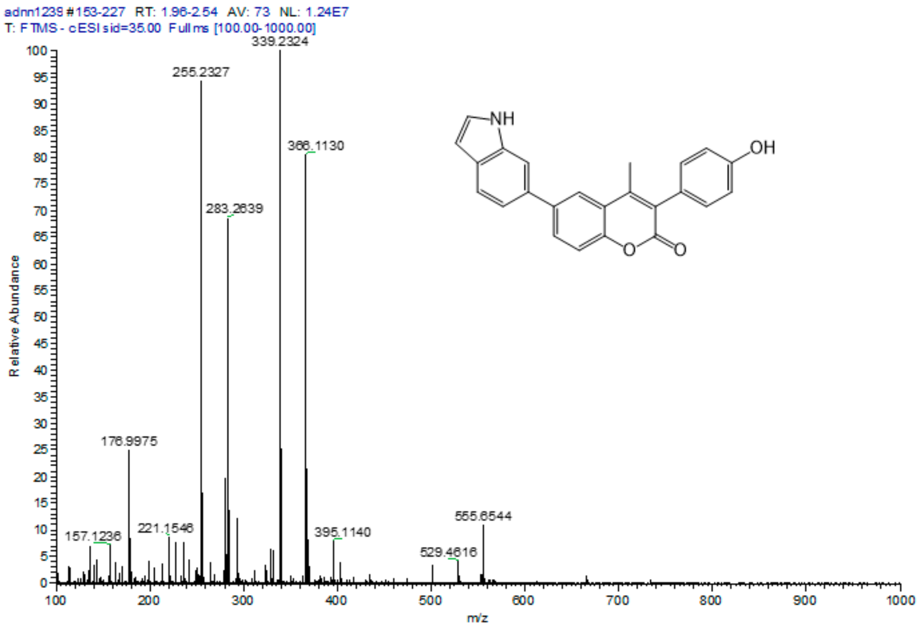

Figure S40. HR-MS spectrum of **4j**.
